# Supplementary material for: Earth‐Based Transmitters Trigger Precipitation of Inner Radiation Belt Electrons: Unveiling Observations and Modeling Results
Source: AGU Adv. 2024 Nov 21;5(6):e2024AV001354. doi: 10.1029/2024AV001354 (PMC11600777; doi:10.1029/2024AV001354)
Supplement: Supplementary file 1 — Supporting Information S1 [file AGA2-5-0-s008.docx]

*AGU Advances*

Supporting Information for

**Earth-Based Transmitters Trigger Precipitation of Inner Radiation Belt Electrons: Unveiling Observations and Modeling Results**

Zheng Xiang^1^, Xinlin Li^1, 2^, Daniel N. Baker^1^, Yang Mei^1,2^, Declan O’Brien^1,2^,

Benjamin Hogan^1,2^, Hong Zhao^3^, David Brennan^1^, Binbin Ni^4^, Theodore Sarris^1,5^,

and Michael A. Temerin^6^

**^1^** Laboratory for Atmospheric and Space Physics, University of Colorado Boulder, Boulder, CO, USA.

**^2^** Department of Aerospace Engineering Sciences, University of Colorado Boulder, Boulder, CO, USA.

**^3^** Department of Physics, Auburn University, Auburn, AL, USA.

**^4^** Department of Space Physics, School of Electronic Information, Wuhan University, Wuhan, Hubei, China.

**^5^** Department of Electrical Engineering, Democritus University of Thrace, Xanthi, Greece.

**^6^** Retired from Space Sciences Laboratory, University of California, Berkeley, Berkeley, CA, USA.

**Contents of this file**

Figures S1 to S7

Text S1

**Introduction**

This document shows 7 supporting figures. Figure S1-S3 shows pitch angle diffusion coefficients from different resonance orders at distinct energies and L values. Figure S4-S5 show the simulation results with different wave normal angles of transmitter signals. Figure S6 shows Locations of VLF transmitter stations when multiple wisps were observed. Figure S7 shows an example of simulation results in the Drift-Diffusion-Source model. Text S1 explains how the pitch angle diffusion coefficient is calculated.


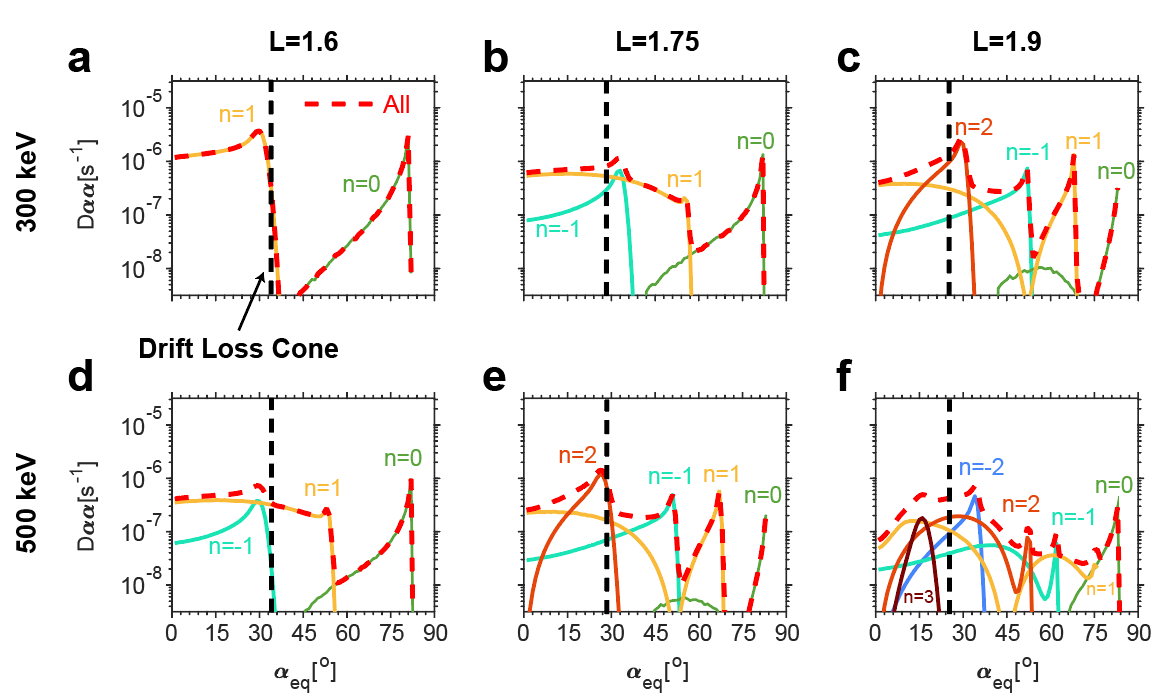


**Figure S1.** Pitch angle diffusion coefficients induced by NWC transmitter signals with 20 pT wave amplitudes and 65˚ centered wave normal angle. Pitch angle diffusion coefficients from different resonance orders are indicated by solid curves with different colors. The summations of difference resonance orders are indicated by the red dashed curves. The vertical black dashed lines represent drift loss cones at the selected L values. There is no diffusion coefficient from n=-3, -2, and 3 since the corresponding resonance energies are much higher.

Figure S1 shows pitch angle diffusion coefficients of 300 keV and 500 keV electrons from different resonance orders (n=-3 to n=3) at L=1.6, 1.75, and 1.9. For 300 keV electrons, n=1, -1, and 2 dominate the diffusion coefficient near drift loss cone at L=1.6, L=1.75, and L=1.9 (panels a-c), respectively. Thus, three wisps are produced at L=1.6-1.9. Diffusion coefficients from n=1, 2 are higher than n=-1. Accordingly, the flux level of second wisp is weaker than those of the first and third wisps. Those features are observed by CIRBE/REPTile-2 (Figure 2 in the main text) and simulated in the Drift-Diffusion-Source model (Figure 3 in the main text). These results demonstrate that harmonics can be differentiated.


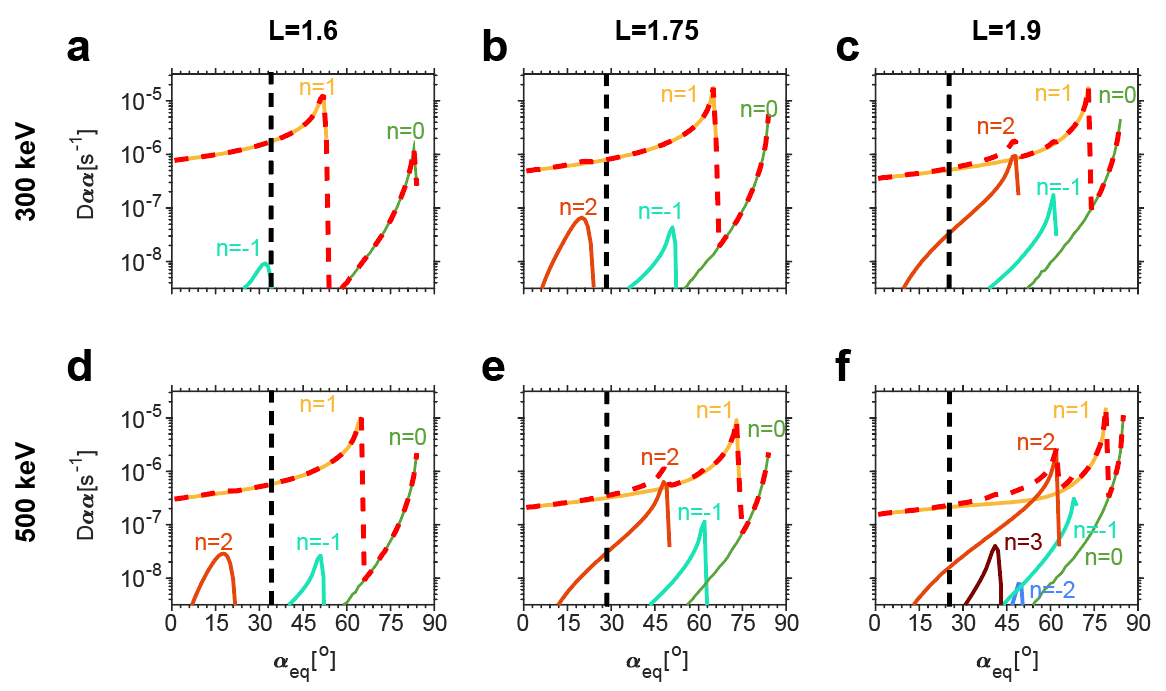


**Figure S2.** Same format as Figure S1 but for 15˚ centered wave normal angle

It can be observed that n=1 dominates the diffusion coefficient near drift loss cone at the three selected L values. As a result, one wisp is generated in the Drift-Diffusion-Source model (see Figure S5 in the supporting information).


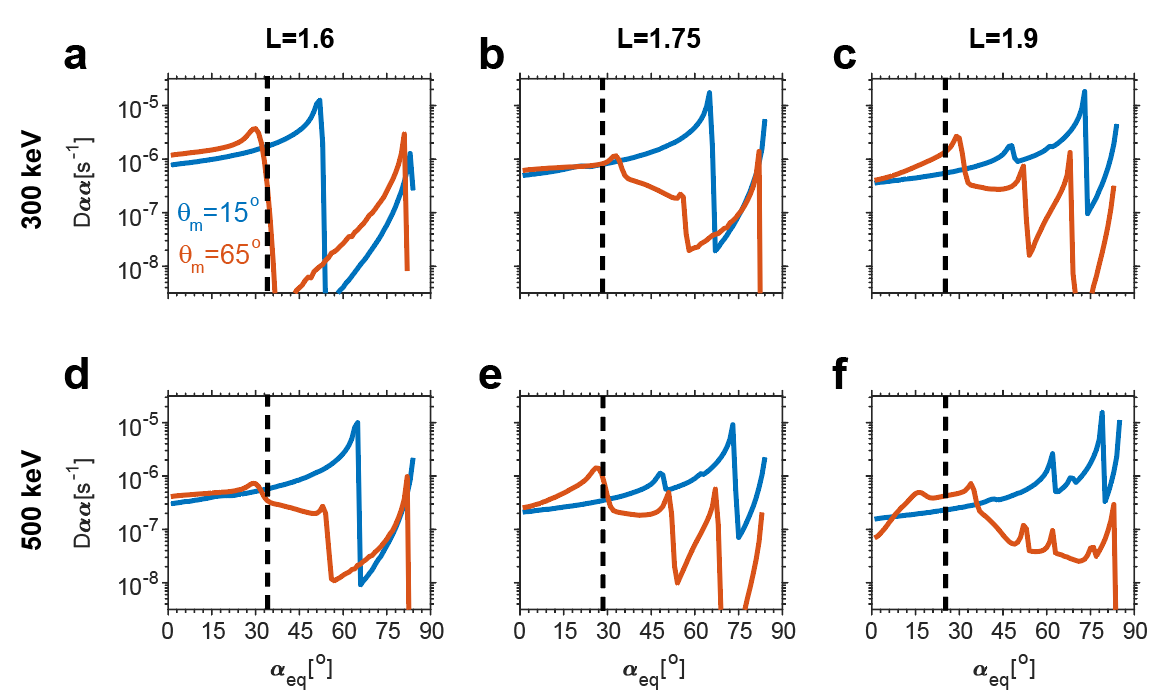


**Figure S3.** Comparison between pitch angle diffusion coefficients induced by parallel (15˚ centered wave normal angle) and oblique (65 ˚ centered wave normal angle) transmitter signals. Harmonics from n=-3 to n=3 are included.

Diffusion coefficients induced by transmitter signals with small wave normal angles are higher than those induced by highly oblique transmitter signals at larger pitch angles. However, close to the drift loss cones, the diffusion coefficients induced by oblique transmitter signals are larger, especially at higher L values (panels c, e, f). Increasing the wave normal angles do not reduce the diffusion coefficients accounting for the formation of wisps. Thus, the multiple wisps generated by oblique transmitter signals have comparable/higher flux levels than the single wisps induced by parallel transmitter signals.


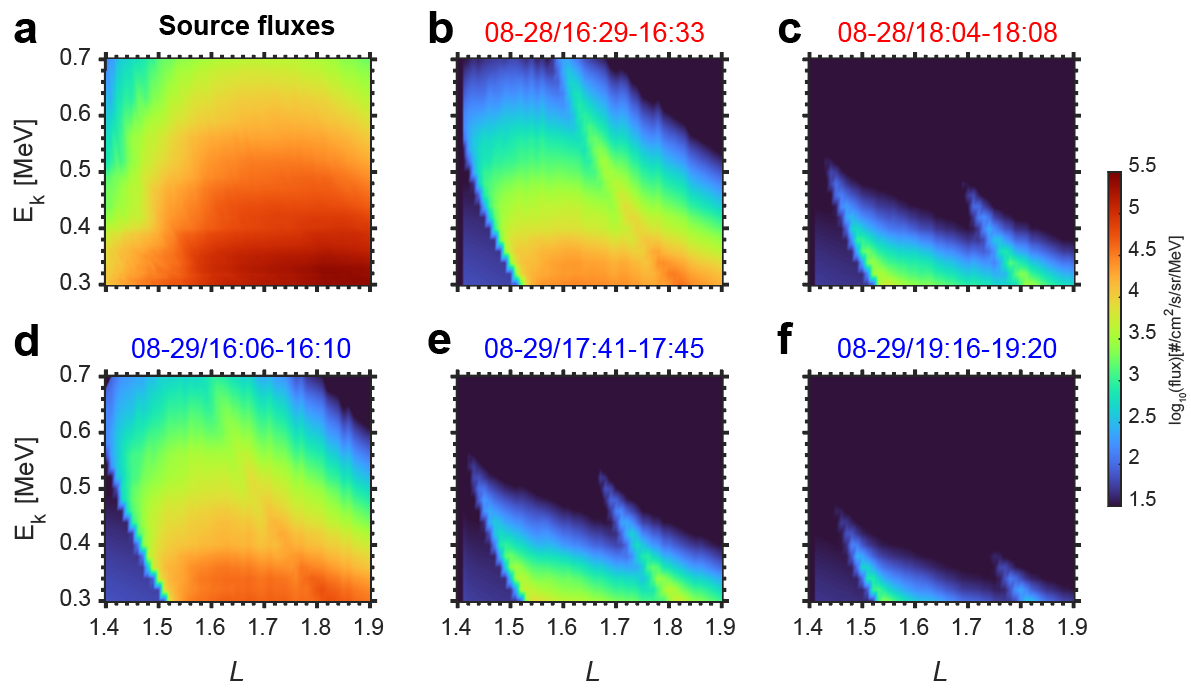


**Figure S4.** Simulation results of multiple wisps using the Drift-Diffusion-Source model with 40˚ center wave normal angle. (a) Source fluxes used in the model to produce quasi-trapped electron fluxes scattered by NWC transmitter signals. (b-f) Simulated electron fluxes correspond to Figure 2b-f. Scattering effects from N=±1,2 resonance orders are included in the simulations.

Figure S4 shows simulation results of multiple wisps with 40˚ center wave normal angle. It can be observed that wisps shift to lower energies compared to the results shown in Figure 3 with a 65° center wave normal angle. The wisp produced by N=-1 resonance is weaker to be observed than the one in Figure 3, suggesting that lower wave normal angles lead to lower resonance energies and weaker diffusion coefficients from N=-1.


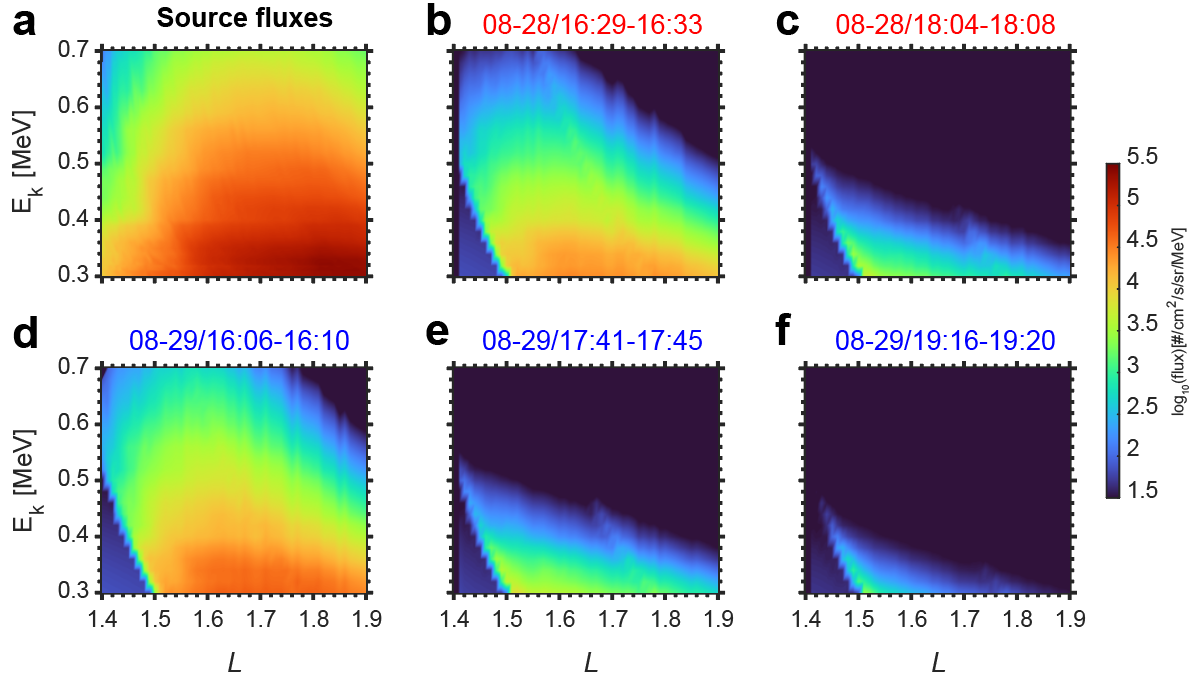


**Figure S5.** same format as **Figure S4** but for simulation results with 15˚ center wave normal angle of NWC transmitter signals.

Figure S5 shows simulation results of multiple wisps with 15˚ center wave normal angle. It can be observed that wisps from N=1 and N=2 are overlapped. The multiple-wisps phenomenon almost disappears in the simulations.


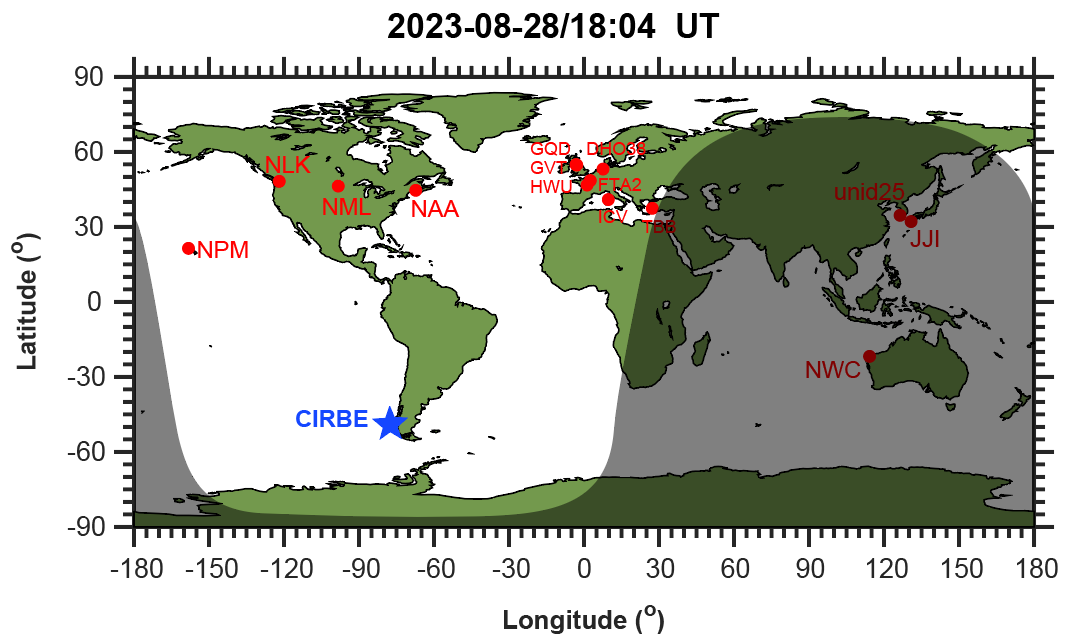


**Figure. S6**. Locations of VLF transmitter stations at the given time epoch. The shadow regions indicate the nightside when the multiple wisps were observed by CIRBE at 18:04 UT on 28 Aug 2023. The blue star indicates the CIRBE position.

Supplementary Figure S6 shows the locations of VLF transmitter stations around the world when multiple wisps were observed on 28 August 2023. The nightside is indicated by the shadow regions using data from https://www.timeanddate.com /astronomy/moon/light.html. It can be observed that most VLF transmitter stations were on the dayside at the given time epoch. Only NWC, JJI, and unid25 stations were on the nightside. The power of JJI and unid25 are much lower than NWC. In addition, the transmitted frequencies of JJI and unid25 are higher than NWC. Waves with higher frequencies resonate with electrons at lower energies (see equation (1)). Thus, they can only produce wisps with energies lower than the wisps produced by NWC. But the observed multiple wisps have two wisps with energies higher than the one with larger flux levels. Therefore, the observed multiple wisps are more likely to be produced by the NWC station by harmonic cyclotron resonances.


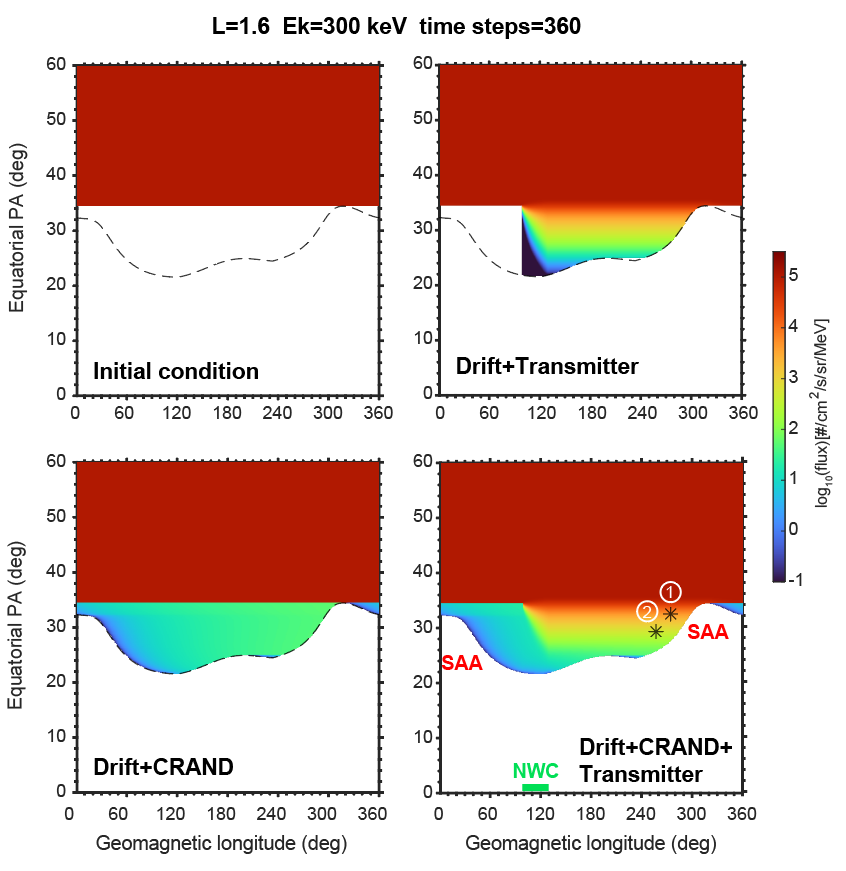


**Figure S7**. Simulation results of 300 keV electrons at L=1.6 including different physical mechanisms. The simulated electron fluxes are plotted as a function of equatorial pitch angles and longitudes. In this coordinate, electrons drift from left to right and diffuse from higher equatorial pitch angels (PA) to lower ones. The SAA is around [300˚, 20˚] longitude range characterized by larger bounce loss cones indicated by blank space extending to higher equatorial PAs. The longitude range adding pitch angle diffusion coefficients induced by NWC transmitter signals is indicated by the green bar at the bottom of this figure. Electron fluxes corresponding to #1 and #2 snow symbols represent simulation results in Figure 3b and 3c, respectively.

Figure S7 shows simulation results of 300 keV electrons at L=1.6 in the Drift-Diffusion-Source model. In the simulation, one time step equals the time for electrons to drift one degree in geomagnetic longitude. Thus, 360 steps indicate one drift period. Electrons drift from the left to right in the figure. After drifting into the regions influenced by NWC transmitter signals (longitudes [99˚, 129˚]), trapped electrons are scatted into the drift loss cone. Then the quasi-trapped electron fluxes increase and drift into the SAA. Thus, quasi-trapped electron fluxes at longitudes>130˚ are all enhanced. The position of #1 and #2 snow symbols are calculated based on the satellite position and IGRF magnetic field model. The local pitch angels of electrons are assumed as 90˚. The detailed calculation process can be found in Xiang et al. (2019). It can be observed that #1 snow symbol is closer to the SAA and has larger equatorial pitch angles than #2 snow symbol. As a result, the simulated fluxes in Figure 3b are higher than those in Figure 3c. The similar simulations are conducted for every 0.01 L at L=1.4-1.9 and every 20 keV at 300-700 keV. Plotting these simulation results as a function of energies and L values obtains the multiple wisps in Figure 3b-f.

**Supplementary Text S1: Pitch angle diffusion coefficient calculation**

Several approaches can be used to simulate wave-particle interactions, e.g. quasi linear theory, Particle-In-Cell, and test particle simulations. In this study, we mainly use quasi linear theory to evaluate scattering effects induced by NWC transmitter signals on inner belt electrons. Based on quasi-linear theory, waves are considered as rapid variations with small amplitudes and resonant wave-particle interactions can be described in terms of pitch angle and energy diffusion coefficients (Kennel & Engelmann, 1966; Lyons et al., 1971, 1974). The cyclotron resonance condition between NWC transmitter signals and electrons can be expressed as:

$\omega-k_{\parallel}v_{\parallel}=N\frac{\Omega}{\gamma}$ (1)

where 𝜔 is wave angular frequency, 𝑘_∥_ is the parallel components of wave number, 𝑣_∥_ is the parallel components of the electron velocity, 𝛾 is the corresponding Lorentz factor, Ω is the electron gyrofrequency, and N = 0, ±1, ±2… which represents the orders of the resonance.

The relation between 𝜔 and 𝑘 is:

$k=\frac{\omega\mu}{c}$ (2)

where $\mu$ is the refractive index and $c$ is the speed of light.

Combing equation (1) and (2) we get:

$\omega(1+\beta\mu\cos\theta\cos\alpha)=N\Omega{(1-\beta^{2})}^{1/2}$ (3)

where $\beta=v/c$, $\frac{1}{\gamma}={(1-\beta^{2})}^{1/2}$, the value range of $\beta$ is [-1,1], when N is positive (negative) $\beta$ is also positive (negative). $\theta$ is wave normal angle, and $\alpha$ is local pitch angle.

Based on the dispersion relations for whistler mode waves, $\mu$ is given by:

$\mu=\left\{ 1+{\omega_{pe}}^{2}/\left[ \omega\left( \Omega\cos\theta-\omega\right) \right] \right\}^{1/2}$ (4)

where $\omega_{pe}=$ ${(n_{e}e^{2}/m_{e}/\epsilon_{0})}^{1/2}$is plasma frequency, $n_{e}$ is the background electron density, $e$ is the electric charge, $m_{e}$ is the electron rest mass, and $\epsilon_{0}$ is the permittivity of free space.

In our case, $\omega$ is 19.8 kHz, $\omega_{pe}$ can be calculated using a background electron density model (Ozhogin et al., 2012) $n_{e}=1.5*{10}^{4.4693-0.4903\cdot L}$, $\Omega$ can be calculated based on a dipole magnetic field, wave normal angle $\theta$ is set as 65˚. We set the resonance order N=1, electron pitch angle $\alpha=10˚$, then the $\mu$ value can be solved based on equation (4):


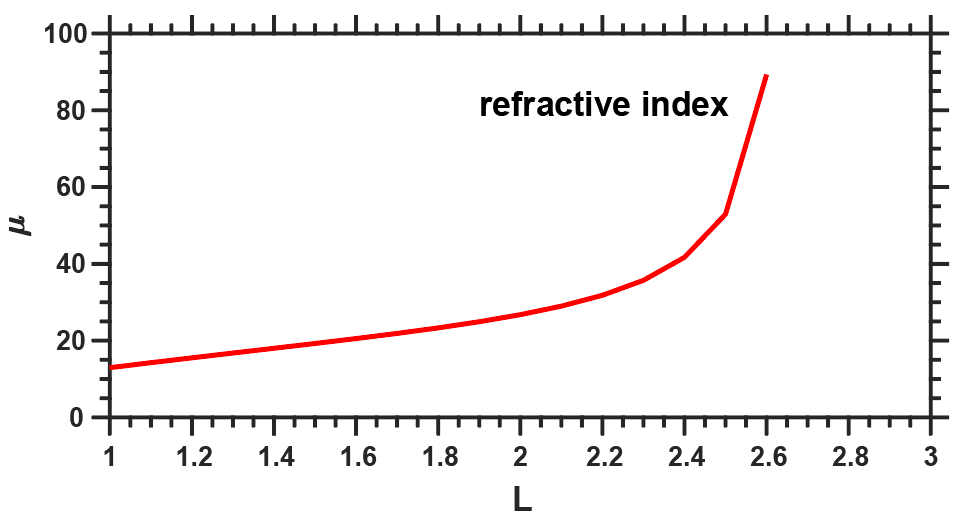


The $\beta$ value obtains from equation (3). The $\beta$ can be used to obtain electron kinetic energies by:

$\gamma={(1-\beta^{2})}^{-1/2}$ (5)

$E_{k}=m_{e}c^{2}\gamma$ (6)

The red solid line shown in the below figure indicates results from equation (6). It can be observed that resonant energies decrease at higher L values.


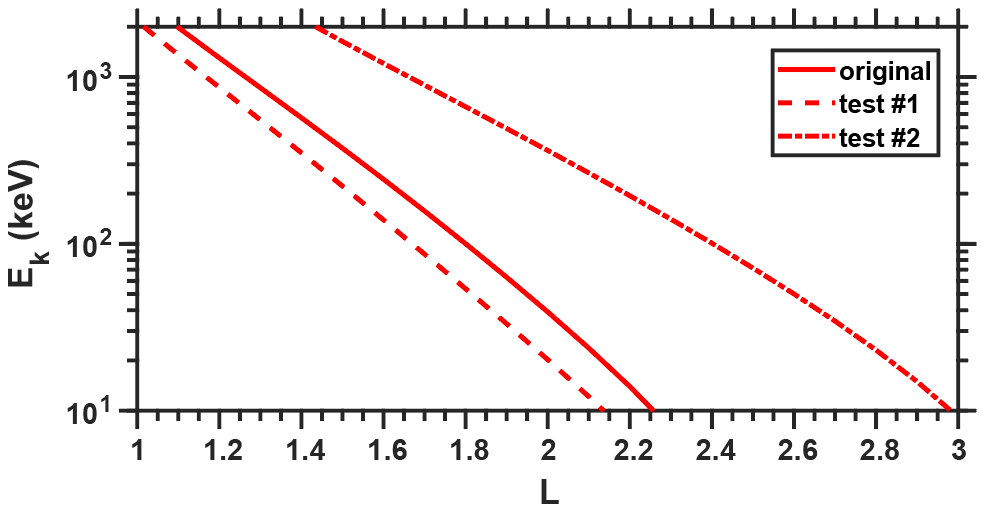


The resonant energies are different at distinct L values due to different background electron densities and magnetic field strength. Thus, we investigate the influences of background electron densities and magnetic field strength on resonant energies. For test #1, the background electron densities are 2 times larger than the original case. For test #2, the background magnetic field strength is 2 times larger than the original case. Other parameters are the same as the original results. It can be observed that stronger magnetic field strength (test #2) leads to higher resonant energies, while larger background electron densities (test #1) lead to lower resonant energies. In addition, the influence of magnetic field strength on resonant energies is more significant than electron densities. For the original results, when L shifts from L=3 to L=1, both background magnetic field strength and electron densities increase. Since magnetic field strength increases more than electron densities (the ratio of magnetic field strength at L=1 and L=3 is 27 while the ratio for electron densities is ~9.5) and has a stronger influence on resonant energies, the resonant energies increase from L=3 to L=1.

When considering the resonance occurring off the equator, the background electron densities are given by:

$n_{e}=1.5*{10}^{4.4693-0.4903\cdot L}*\left[ \cos\left( \frac{\pi}{2}\cdot\frac{\lambda}{\lambda_{INV}} \right) \right]^{-0.75}$ (7)

where $\lambda$ is latitude, and $\lambda_{INV}$ is invariant latitude (where a particular magnetic field line touches the surface of the Earth) calculated by $\lambda_{INV}=arccos\left( \sqrt{1/L} \right)$. The background magnetic field strength still follows the dipole model.

$B=\frac{3.12\times{10}^{4}}{L^{3}}*\frac{\left( 1+3\sin^{2} \lambda\right)^{1/2}}{\cos^{6} \lambda}$ (8)

The background magnetic field strength and electron density along the field line at L=1.7 are shown below:


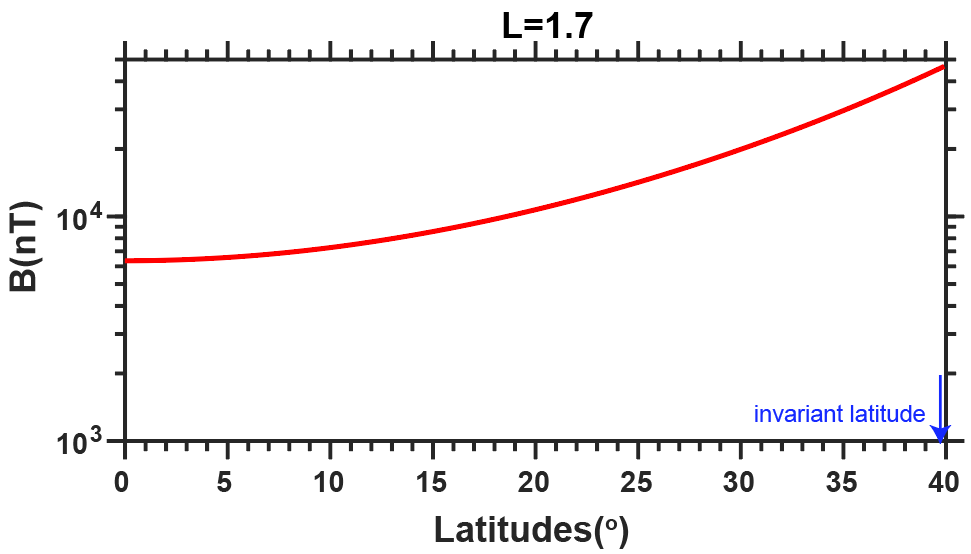


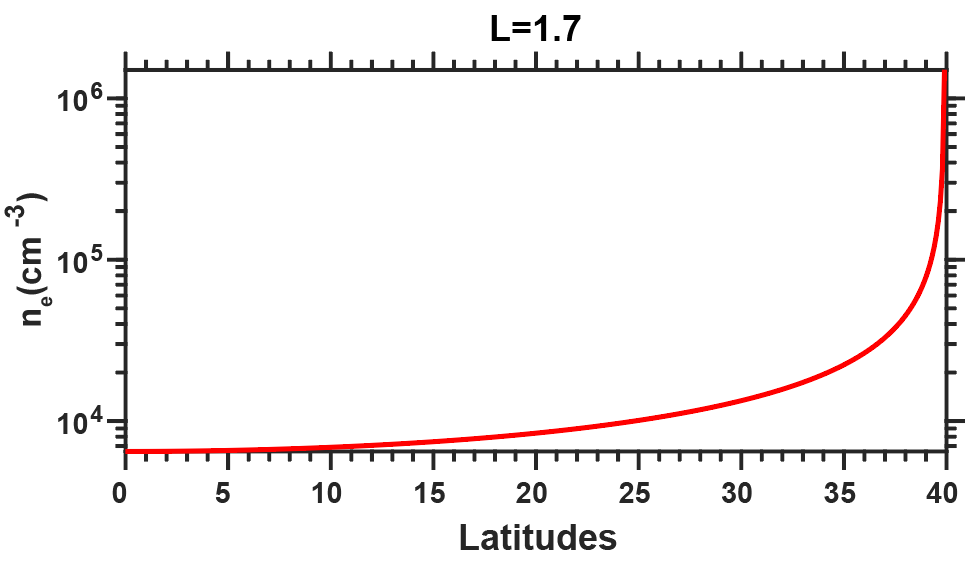


Wave frequency of transmitter signals is set as 19.8 kHz and first resonance order N=1 is considered. The resonant energies of electrons with 10˚ pitch angle at L= 1.7 as a function of latitudes and wave normal angles are shown below:


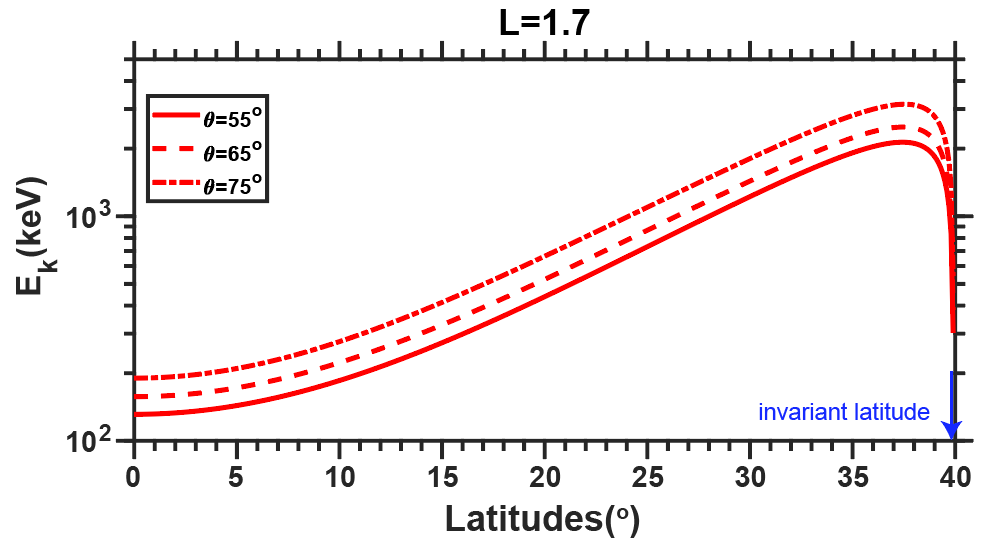


Resonance energies increase as latitudes increase and then decrease when close to the invariant latitude due to the sudden increase of background electron densities close to the surface of Earth. For 300-700 keV electrons simulated in this study, the wave-particle cyclotron interaction mainly occurs at <20˚ latitudes.

When the resonance condition equation (1) is satisfied, the local pitch angle coefficient can be calculated by (Albert, 2007):

$\frac{D_{\alpha\alpha}}{p^{2}}=\frac{\Omega}{\gamma^{2}}\frac{B_{wave}^{2}}{B_{0}^{2}}\sum_{N=-\infty}^{\infty} \sum_{\omega} D_{\alpha\alpha}^{N}$ (9)

with

$D_{\alpha\alpha}^{N}=\int_{\theta_{min}}^{\theta_{max}} \sin\theta d\theta\Delta_{N}G_{1}G_{2}$ (10)

$\Delta_{N}\left( \omega,\theta\right)=\frac{\pi}{2}\frac{\sec\theta}{\left| {v_{\parallel}}/c \right|^{3}}\Phi_{N}^{2}\frac{\left( -\sin^{2} \alpha+N\Omega/\omega\gamma\right)^{2}}{\left| 1-{\left( \partial\omega/k_{\parallel} \right)_{\theta}}/{v_{\parallel}} \right|}$ (11)

$\Phi_{N}^{2}=\left[ \left( \frac{D}{\mu^{2}-S} \right)^{2}\left( \frac{\mu^{2}\sin^{2} \theta-P}{\mu^{2}} \right)^{2}+\left( \frac{P\cos\theta}{\mu^{2}} \right)^{2} \right]^{-1}$

$\times\left[ \frac{\mu^{2}\sin^{2} \theta-P}{2\mu^{2}}\left( 1+\frac{D}{\mu^{2}-S} \right)J_{N+1}+\frac{\mu^{2}\sin^{2} \theta-P}{2\mu^{2}}\left( 1-\frac{D}{\mu^{2}-S} \right)J_{N-1}+\cot\alpha\sin\theta\cos\theta J_{N} \right]^{2}$ (12)

$G_{1}(\omega)=\frac{\Omega B^{2}(\omega)}{\int_{\omega_{LC}}^{\omega_{UC}} B^{2}(\omega^{'})d\omega^{'}}$ (13)

$G_{2}(\omega,\theta)=\frac{g_{\omega}(\theta)}{N(\omega)}$ (14)

$N(\omega)=\int_{\theta_{min}}^{\theta_{max}} d\theta^{'}\sin\theta^{'}\Gamma g_{\omega}(\theta')$ (15)

$\Gamma=\mu^{2}\left| \mu+\omega\frac{\partial\mu}{\partial\omega} \right|$ (16)

In these formulas, $p$ is electron momentum, $B_{wave}$ is wave amplitude, $D, S, P$ are the Stix parameters, $J_{N}$ is Bessel functions, $B^{2}\left( \omega\right)=B_{0}^{2}e^{-\left( \omega-\omega_{m} \right)^{2}/\delta\omega^{2}}$ describe the frequency distribution of wave power, $B_{0}$ ensures that ${\int B^{2}\left( \omega\right)d\omega=B}_{wave}^{2}$ , $g_{\omega}(\theta)$ describe the distribution of wave power with wave normal angle. In this study, VLF waves are assumed to have a Gaussian frequency distribution, centered at the NWC transmitter frequency 19.8 kHz with a bandwidth of ±100 Hz, namely$\omega_{LC}=19.7*2\pi kHz, \omega_{UC}=19.9*2\pi kHz,\omega_{m}=19.8*2\pi kHz,\delta\omega=0.1*2\pi kHz$ . The wave amplitude is set as 20 pT. The wave normal angle also follows a Gaussian distribution and is set as θ_min_=55˚, θ_max_=75˚, θ_m_=65˚, and θ_w_=10˚.

Local pitch-angle diffusion coefficients are time averaged over an electron bounce-orbit to get the bounce-averaged pitch-angle diffusion coefficients $\left\langle D_{\alpha_{eq}\alpha_{eq}} \right\rangle$ given by (Summers et al., 2007):

$\left\langle D_{\alpha_{eq}\alpha_{eq}} \right\rangle=\frac{1}{T\left( \alpha_{eq} \right)}\int_{0}^{\lambda_{m}} D_{\alpha\alpha}\frac{\cos\alpha}{\cos^{2} \alpha_{eq}}\cos^{7} \lambda d\lambda$ (17)

where $T\left( \alpha_{eq} \right)=1.3-0.56\sin\alpha_{eq}$ is the normalized bounce time, $\lambda$ is the magnetic latitude and $\lambda_{m}$ is the latitude of the mirror point of the particle. The $\left\langle D_{\alpha_{eq}\alpha_{eq}} \right\rangle$ are used in the Drift-Diffusion-Source model to reproduce multiple wisps observed by CIRBE/REPTile-2.
