# Supplementary material for: Earth‐Based Transmitters Trigger Precipitation of Inner Radiation Belt Electrons: Unveiling Observations and Modeling Results
Source: AGU Adv. 2024 Nov 21;5(6):e2024AV001354. doi: 10.1029/2024AV001354 (PMC11600777; doi:10.1029/2024AV001354)
Supplement: Supplementary file 2 — Original Version of Manuscript [file AGA2-5-0-s002.pdf]

Earth-Based Transmitters Trigger Precipitation of Inner Radiation Belt Electrons: Unveiling Observations and Modeling Results

Zheng Xiang[1], Xinlin Li[1, 2], Daniel N. Baker[1], Yang Mei[1,2], Declan O'Brien[1,2], Benjamin Hogan[1,2], Hong Zhao[3], David Brennan[1], Binbin Ni[4], Theodore Sarris[1,5], and Michael A. Temerin[6]

[1] Laboratory for Atmospheric and Space Physics, University of Colorado Boulder, Boulder, CO, USA.

[2] Department of Aerospace Engineering Sciences, University of Colorado Boulder, Boulder, CO, USA.

[3] Department of Physics, Auburn University, Auburn, AL, USA.

[4] Department of Space Physics, School of Electronic Information, Wuhan University, Wuhan, Hubei, China.

[5] Department of Electrical Engineering, Democritus University of Thrace, Xanthi, Greece.

[6] Retired from Space Sciences Laboratory, University of California, Berkeley, Berkeley, CA, USA.

*Correspondence to: xinlin.li@lasp.colorado.edu

Key Points:

- Multiple wisps induced by NWC transmitter signals were captured by CIRBE/REPTile-2.
- Simulations reproduce multiple wisps by including 1, -1, and 2 cyclotron resonance orders.
- Highly oblique transmitter signals are necessary to produce multiple wisps.

Abstract

Human activity influences Earth's environment, including the space environment hundreds to thousands of kilometers above the Earth. One direct evidence is that the 19.8 kHz electromagnetic signals launched by the North West Cape (NWC) transmitter station in Australia produce a wisp-like energy distribution of precipitating energetic electrons in Earth's inner radiation belt, observed by many Low Earth Orbiting (LEO) satellites. Typically, satellites observe a single wisp with energy that decreases with increasing L (approximately the radial distance in the equatorial plane), which is produced by a first-order cyclotron resonance of transmitter signals with energetic electrons. Here we report, for the first time, multiple wisps observed by the Relativistic Electron and Proton Telescope integrated little experiment-2 (REPTile-2) on board the recently launched Colorado Inner Radiation Belt Experiment (CIRBE) CubeSat. Based on simulation results, we demonstrate that harmonic cyclotron resonances including the 1, -1, and 2 orders by highly oblique NWC transmitter signals produce these multiple wisps. The discovery of multiple-order cyclotron resonances simultaneously occurring in space sheds new light on wave-particle interactions in near-Earth space. It also has implications for developing artificial radiation belt remediation techniques and understanding the propagation and scattering of plasma waves in planetary magnetospheres.

Plain Language Summary

To communicate with submarines, very low frequency (VLF, 3-30 kHz) transmitter stations were built around the world since their signals can penetrate seawater to a few tens of meters. Generally, these VLF transmitter signals propagate within the Earth-ionosphere waveguide. On the nightside, electron densities in the ionosphere are weak, some transmitter signals can leak into the magnetosphere and interact with energetic electrons through cyclotron resonances. Previous satellite observations suggest that the cyclotron resonance between transmitter signals and energetic electrons is mostly first-order and produces a wisp-like energy spectrum of precipitation electrons. Thanks to the high- energy and time resolution of the REPTile-2 instrument, multiple wisps were captured and reproduced by simulations with harmonic resonance orders (-1, 1, and 2). The scattering effect induced by space plasma waves from different resonance orders has never been examined by observation until our present work. Energetic electrons in Earth's radiation belts have detrimental effects on spacecraft subsystems and are harmful to astronauts during extravehicular activity. Scattering by the high-power VLF waves has been considered a promising method for human control of Earth's radiation belts. Our results demonstrate that oblique human-made VLF waves can scatter electrons in a wider energy range through harmonic cyclotron resonances. This sheds new light on developing artificial radiation belt remediation techniques to mitigate radiation hazards on spacecraft and astronauts.

1. Introduction

Energetic electron fluxes in Earth's inner radiation belt increase during active geomagnetic times followed by slow decay during quiet periods (Selesnick, 2015; Xiang et al, 2020a). Their energy spectra can often be characterized by exponential distributions (Mihalov & White 1966; Zhao et al., 2019a). Due to atmospheric collisions and wave-particle interactions, trapped inner belt electrons can be scattered into the drift loss cone and precipitate after encountering the South Atlantic Anomaly (SAA), where the weaker magnetic field leads to an altitude decrease of the electron's mirror point (Selesnick et al., 2003; Tu et al., 2010). The electrons in the drift loss cone are called quasi-trapped since their lifetime is shorter than one drift period, which depends on the energy and L of the electrons (e.g., a 500 keV electron at L=1.2 has a drift period of 1.5 hours). Different from trapped electrons, energy spectra of quasi-trapped inner belt electrons typically follow a CRAND (Cosmic Ray Albedo Neutron Decay) - produced spectrum discovered by measurements from CSSWE/REPTile and DEMETER/IDP (Selesnick, 2015; Li et al, 2017; Zhang et al., 2019; Xiang et al, 2019). However, peaks in the energy spectra of quasi-trapped electrons are frequently observed at L≈1.4-1.8 (Vampola & Kuck, 1978; Datlowe, 2006; Liu et al., 2022). The energy of the peaks decreases rapidly with increasing L. They were first observed by satellite 1971 089A (Imhof et al., 1973) and were first called a "wisp" to describe enhancements of quasi-trapped electrons induced by NWC transmitter observed by DEMETER/IDP (Sauvaud et al., 2008).

Energetic electrons trapped by Earth's magnetic field experience three periodic motions: gyration around a magnetic field line, bounce along a magnetic field line between mirror points, and azimuthal drift around the Earth. Associated with each periodic motion, plasma waves with similar frequencies (including Doppler-shifted effects) can resonate with energetic electrons,

leading to changes of their energy, pitch angle, and L, accounting for the dynamics of Earth's radiation belts (Baker et al., 2013, 2014; Thorne et al., 2013; Shprits et al., 2013, 2016; Mann et al., 2016; Zhao et al., 2019). One common and important wave-particle interaction in Earth's radiation belts is cyclotron resonance induced by whistler mode waves, producing both acceleration and precipitation of energetic electrons (Roth et al., 1999; Horne et al., 2005; Thorne et al., 2010; Kasahara et al., 2018). Whistler mode waves such as chorus waves and plasmasphreic hiss can be naturally generated (Meredith et al., 2006; Bortnik et al., 2008; Omura et al., 2008; Teng et al., 2023). Human-made Very-Low-Frequency (VLF) electromagnetic signals leaking into near-Earth space also propagate in the whistler mode and can interact with energetic electrons (Koons et al., 1981; Sauvaud et al., 2008). One direct observational evidence is the wisp spectrum observed by LEO satellites in Earth's inner belt (Liu et al., 2022; Shen et al., 2022). Previous theoretical and observational studies demonstrated that first order cyclotron resonance of NWC transmitter signals with energetic electrons can account for the formation of wisps (Gamble et al., 2008; Selesnick et al., 2013).

The cyclotron resonance condition between NWC transmitter signals and electrons can be expressed as (Koons et al., 1981):

$$\omega - k_{\parallel} v_{\parallel} = N \frac{\Omega}{\gamma}, \qquad (1)$$

where ω is wave angular frequency, $k_{\parallel}$ is the parallel component of the wave number, $v_{\parallel}$ is the parallel component of the electron velocity, γ is the Lorentz factor, Ω is the electron gyrofrequency, and N = 0, ±1, ±2… represent the order of the resonance with N=0 corresponding to the Landau resonance. We can see from equation (1) that $v_{\parallel}$ and thus energy must decrease with increasing L, that is, with decreasing Ω since $k_{\parallel}$ changes slowly with increasing L. For parallel propagating waves only the N=1 order is effective. But for energetic electrons and

obliquely propagating waves where the electron gyroradius is comparable to the perpendicular wavelength, the other orders can be effective in pitch-angle scattering energetic electrons. For whistler mode waves, a positive resonance order means the direction of motion of waves and electrons are opposite, while a negative resonance order means the same direction. Different resonance orders produce distinct scattering effects on electrons, which have been theoretically studied for many years (Lyons et al., 1971, 1974; Ni et al., 2011). However, the exact scattering effects from different resonance orders have never been examined by observations. For naturally generated waves, the scattering effects from different orders are mixed up due to their wide frequency range. In contrast, the nearly monochromatic NWC transmitter signal, bandwidth 19.8±0.1 kHz, allows scattering effects from different orders to be clearly distinguished. In this work, we report, for the first time, multiple wisps observed by CIRBE/REPTile-2 and experimentally verify scattering induced by different resonance orders.

Figure 1 illustrates the formation of wisps observed by CIRBE/REPTile-2. Harmonic resonances between NWC transmitter signals and energetic electrons produce single, double, and multiple wisps close to the location of the NWC station. These scattered electrons drift eastward due to the gradient and curvature of Earth’s magnetic field and precipitate into the SAA where the weak magnetic field lowers the mirror point of the electrons. CIRBE/REPTile-2 observed these scattered electrons on the dayside with single, double, and multiple wisps due to scattering effects by N=1, N=±1, and N=±1,2 cyclotron resonances, respectively. The wisps from N=1 and N=-1 partly overlap. Thus, fine energy and time resolution is needed to distinguish wisps from N=1 and N=-1. This is likely why multiple wisps were overlooked by previous satellite measurements but captured by CIRBE/REPTile-2. When the NWC station is on the nightside and turns on, CIRBE/REPTile-2 almost always captures wisps to the west of the SAA. From the

beginning of the mission to the end of year 2023 (256 days) within longitude range [-140°, -70°] in the southern hemisphere, CIRBE/REPTile-2 observed 105 single wisp events (59.7%), 22 double wisp events (12.5%), and 49 multiple wisp events (27.8%). The observed single- and multiple-wisp features are successfully reproduced in the simulation including different resonance orders with NWC transmitter signal, unveiling that the driving mechanism of multiple wisps are multiple harmonic cyclotron resonances.

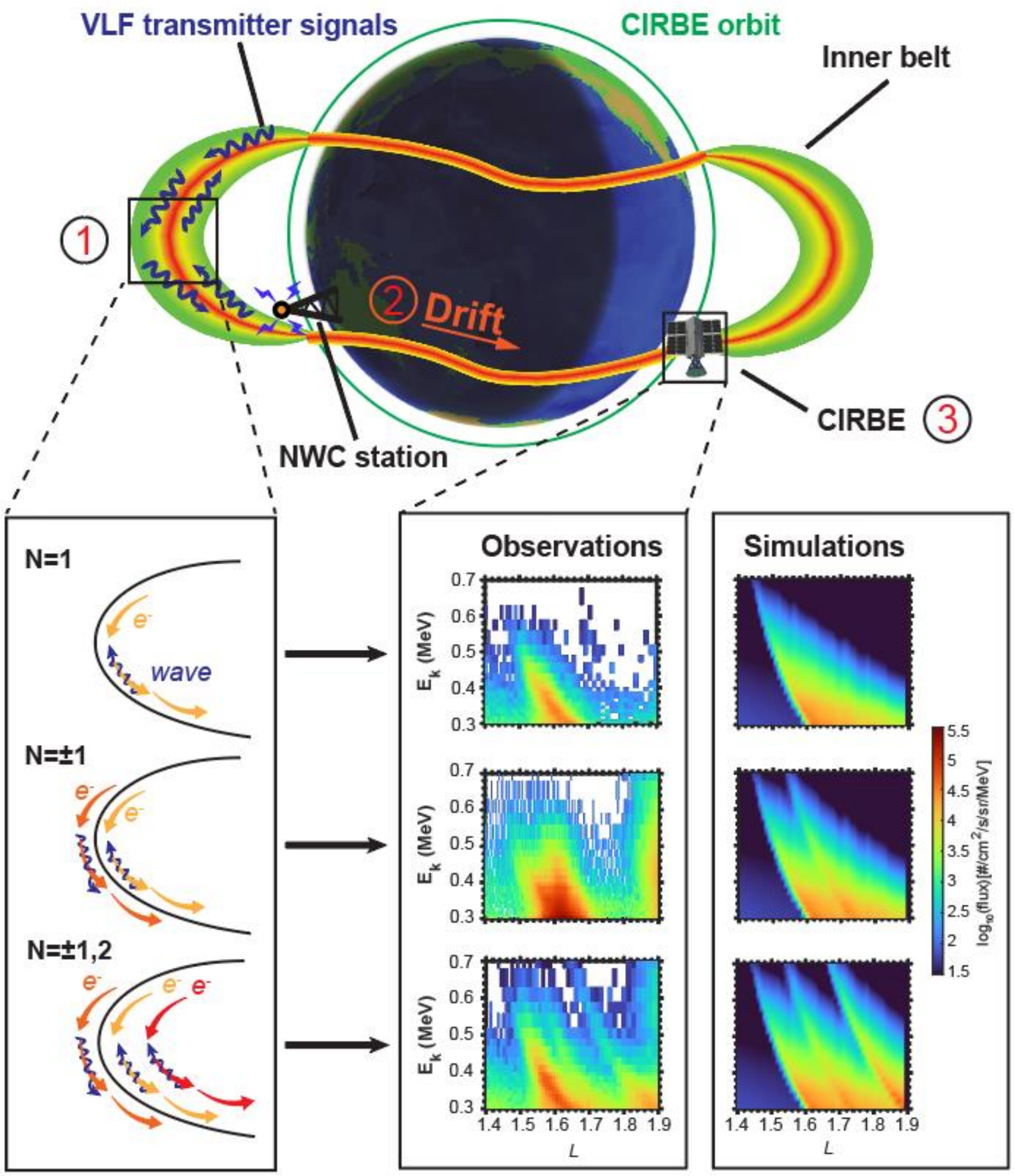

Figure 1. *Schematic illustration of multiple wisps produced by harmonic cyclotron resonances of NWC transmitter signals with energetic electrons. On the nightside, signals launched by the ground based NWC station penetrate the ionosphere and leak into the magnetosphere. The thicker ionosphere on the dayside better shields the radiation belt from the NWC signal. These signals bounce between the northern and southern hemispheres to become highly oblique. The interaction between NWC transmitter signals and energetic electrons occurs at different energies through distinct resonance orders. For resonance order N=1, the direction of motion of waves and electrons is opposite, while their direction is the same for N=-1. Similarly, waves and electrons move in opposite directions for N=2 and the resonance energy is higher. Each resonance order produces a wisp signature in the electron energy spectra. Together, they produce multiple wisps (Step 1). In the zoom-in panel of Step 1, blue arrows indicate waves while yellow, orange, and red arrows indicate electrons with increasing energies. Wave-particle interactions only happen close to the NWC station due to the limited range of leaked signals in space. Then, the scattered electrons drift eastward (Step 2) until they precipitate into the SAA. CIRBE/REPTile-2 on the dayside observes multiple wisps drifting from the night-side (Step 3). The single, double, and triple wisps in the observation panels are from CIRBE/REPTile-2 data at 16:54-16:59 UT on 31 Aug, 16:15-16:20 UT on 15 Dec, and 16:29-16:33 UT on 28 Aug of 2023, respectively. During these periods, the NWC station was on the nightside. Simulations are from the Drift-Diffusion-Source model including the N=1, N=±1, and N=±1,2 resonance orders, respectively.*

2. Observations

We obtained measurements of multiple wisps through the Relativistic Electron and Proton Telescope integrated little experiment-2 (REPTile-2) on board the Colorado Inner Radiation Belt Experiment (CIRBE), a 3U CubeSat launched into a sun synchronous orbit (97.4° inclination, 509 km altitude, with a local time ascending node at 10:30 and a descending node at 22:30) on 15 April 2023 (Li et al., 2022, 2024a, 2024b). REPTile-2 incorporates pulse-height-analysis to enable 60 electron energy channels (0.25-6 MeV) and 60 proton channels (6.5-100 MeV) at a cadence of one second in the SAA regions and five seconds outside (Khoo et al., 2022; Li et al., 2024a). The nominal energy resolution (ΔE/E) is ~10% for the electron energy channels. Guard rings are used to eliminate signals from side penetrating particles. The active attitude control system on CIRBE keeps the pointing direction of REPTile-2 nearly perpendicular to the background magnetic field with a ~51° field of view.

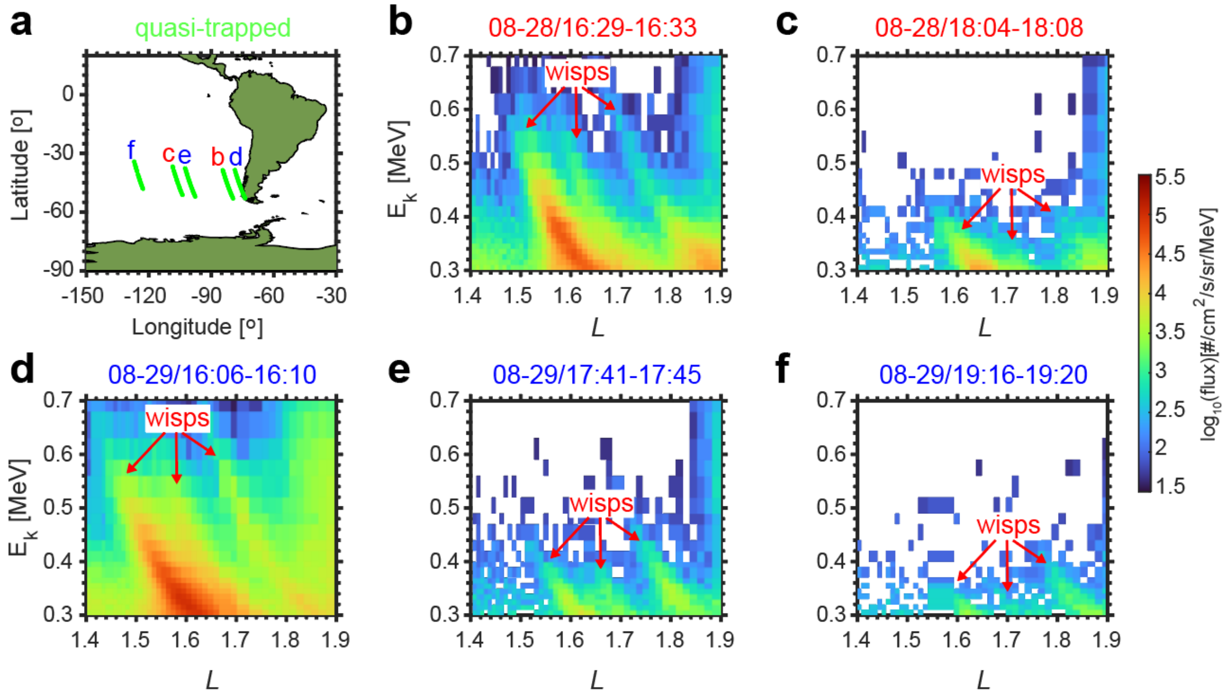

Figure 2. Multiple wisps observed by CIRBE/REPTile-2 on two successive days of 28-29 August 2023. (a) Satellite tracks of the five multiple wisps events in panels (b-f). The measured electrons are all quasi-trapped (with pitch angles larger than the local bounce loss cone but smaller than the drift loss cone, these electrons precipitate into the atmosphere when they drift into the SAA). Red and blue lettering indicate measurements on 28 and 29 August 2023, respectively. (b-f) Electron flux measurements as a function of energy and L, calculated from the IGRF magnetic field model. Three wisps shown in each panel are indicated by red arrows.

Using electron measurements with fine energy and time resolution from CIRBE/REPTile-2, we found five multiple-wisps events during 28-29 August 2023, consisting of three wisps with gaps in between. Geomagnetic activity was quiet during the two days with $Kp \leq 2$ and $Dst \geq -12$ nT. Figure 2a displays the satellite tracks corresponding to the electron fluxes shown in Figure 2b-f. Each satellite track lasted around four minutes during which the NWC station was on the nightside. Electrons were measured to the west of the SAA above the southern hemisphere. All wisps have lower energies at higher L values. The three wisps cover L=1.45-1.65, L=1.6-1.75, and L=1.7-1.9, respectively. Their energies cover 300-600 keV. The first and third wisps have larger fluxes than the second wisp. Flux levels in Figure 2b and Figure 2d are higher since they are measured closer to the SAA where the magnetic field is weaker, and the

observed electrons have higher equatorial pitch angles (see Figure S4). Electron fluxes at higher equatorial pitch angles have larger values (Datlowe, 2006; Shen et al., 2022) since wisps are formed by electrons scattered from the trapped population. For the same reason, wisps seen in the northern hemisphere have lower fluxes because the magnetic field is stronger there.

3. Simulations

We performed numerical simulations to test our hypothesis that multiple wisps are caused by harmonic cyclotron resonance of energetic electrons with NWC transmitter signals. We use the Drift-Diffusion-Source model (Xiang et al., 2019, 2020a, 2020b; Liu et al., 2022) to reproduce observations in Figure 2b-f. Different from the traditional Fokker-Planck simulations adopted for Earth's radiation belts, the Drift-Diffusion-Source model also includes a drift term and uses the IGRF magnetic field model to calculate the bounce loss cone at different longitudes (Selesnick et al., 2003; Tu et al., 2010). Thus, the Drift-Diffusion-Source model can simulate the dynamics of quasi-trapped electrons and have reproduced the longitudinal distributions of quasi-trapped electrons produced by CRAND (Xiang et al., 2019) and NWC transmitter signals (Liu et al., 2022). The equation of the Drift-Diffusion-Source model is given by:

$$\frac{\partial J}{\partial t} + \omega_d \frac{\partial J}{\partial \phi} = \frac{1}{G_0(\alpha_{eq})\sin(2\alpha_{eq})} \frac{\partial}{\partial \alpha_{eq}} \left[G_0(\alpha_{eq})\sin(2\alpha_{eq}) \langle D_{\alpha_{eq}\alpha_{eq}} \rangle \frac{\partial J}{\partial \alpha_{eq}} \right] + S_e, \quad (2)$$

where J is the electron flux, ω_d is the bounce-averaged drift frequency, ϕ is the drift phase or geomagnetic longitude, $G_0(\alpha_{eq}) = v\tau_b/2$ for the bounce period τ_b and the electron speed v, α_{eq} is the equatorial pitch angle, and t is time. S_e is the electron source rate from CRAND which is the main source of quasi-trapped electrons in the inner belt when the NWC station is on the dayside (Liu et al., 2022). $\langle D_{\alpha_{eq}\alpha_{eq}} \rangle$ are bounce-averaged pitch angle diffusion coefficients.

We use a standard fully implicit method to solve equation (2). The grid points in α_{eq} and ϕ are 900×360. The empty bounce loss cone is indicated by $J = 0$ as a boundary condition. The other boundary condition is $\frac{\partial J}{\partial \alpha_{eq}} = 0$ at $\alpha_{eq} = 90°$ for each ϕ. In the simulation, one time step equals the time for electrons to drift one degree in geomagnetic longitude. One simulation example for 300 keV electrons at L=1.6 is shown in Figure S4.

Based on quasi linear theory, the scattering effects induced by NWC transmitter signal can be represented by pitch angle diffusion coefficients (Lyons et al., 1971, 1974; Ni et al., 2011) used in the Drift-Diffusion-Source model. The initial electron flux (Figure 3a) is the trapped electron flux measured by CIRBE/REPTile-2 at 13:23-13:29 UT on 28 August 2023 (~3 hours before the multiple wisps shown in Figure 2b). The variation of trapped electrons in the inner belt is slow during quiet periods. Thus, the same initial flux is used for the simulation in Figure 3b-f. The simulation reproduces the three-wisp features with good agreement with L and energy, demonstrating that multiple wisps are caused by the harmonic cyclotron resonance of electrons with NWC signals. In addition to the wisp positions, flux levels of wisps are also well reproduced in the Drift-Diffusion-Source model. Figure 3d has the highest flux levels while Figure 3f has the lowest flux levels. This explains why CIRBE/REPTile-2 observe fewer wisps far away from SAA regions.

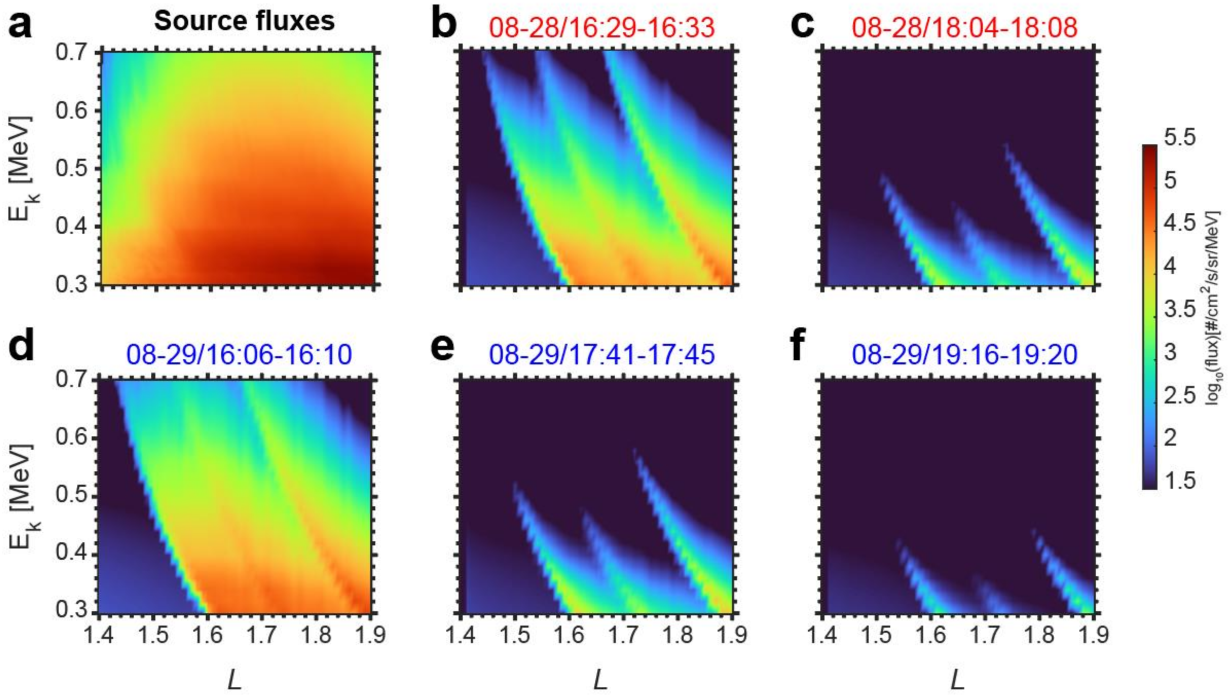

***Figure 3**. Simulation results of multiple wisps using the Drift-Diffusion-Source model. (a) Source flux used in the model to produce quasi-trapped electron fluxes scattered by NWC transmitter signals. (b-f) Simulated electron fluxes correspond to Figure 2b-f. Scattering effects from N=±1,2 resonance orders are included in the simulations.*

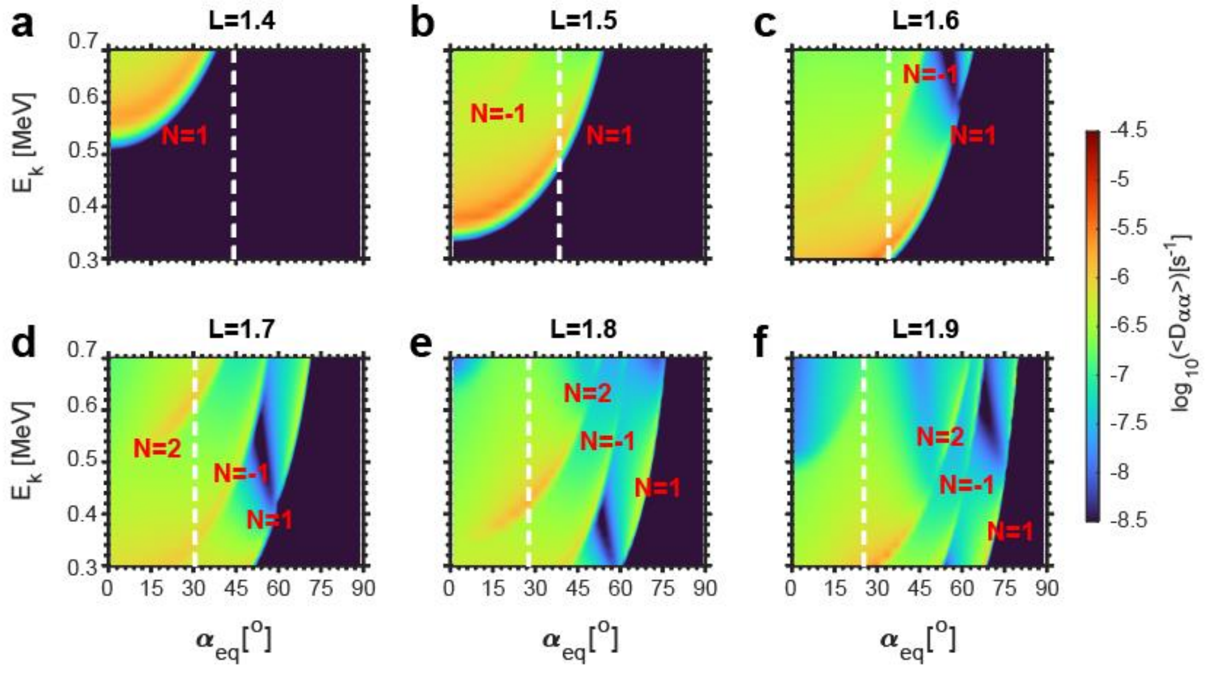

***Figure 4.** 2-D plots of bounce-averaged pitch-angle diffusion coefficients $\langle D_{\alpha_{eq}\alpha_{eq}} \rangle$ as a function of electron kinetic energy and equatorial pitch angle at six selected L values. The drift loss cone*

at the given L is indicated by the white dashed lines. The diffusion coefficients from different resonance orders are indicated.

In the inner belt, since frequencies of NWC transmitter signals are well below the electron gyrofrequency (109.2 kHz at the equator and 1380.9 kHz at the surface of Earth for L=2), and cyclotron interactions mainly produce pitch angle diffusion (Kennel & Engelmann, 1966). The Full Diffusion Code (Ni et al., 2008, 2011) is implemented to compute the bounce-averaged pitch-angle diffusion coefficients using formulas derived by the quasi-linear theory (Albert, 2007), in which the scattering effect of waves on electrons can be modeled as a diffusion equation (Kennel & Engelmann, 1966), with the diffusion coefficients proportional to the wave power. The Full Diffusion Code calculate diffusion coefficients in energy and pitch angle given the magnetic field, plasma density, frequency, and the wave normal angle distribution of waves. It has been previously used to calculate diffusion coefficients induced by naturally generated and human-made whistler mode waves (Thorne et al., 2013; Hua et al., 2020; Xiang et al., 2020b; Liu et al., 2022). To calculate the pitch-angle diffusion coefficients, NWC transmitter signals are assumed to have a Gaussian frequency distribution, centered at the frequency 19.8 kHz with a bandwidth of ±100 Hz. The wave amplitude is set as 20 pT. The wave normal angle distribution is set as θ_{min}=55˚, θ_{max}=75˚, θ_m=65˚, and θ_w=10˚. Considering that the refraction index approaches infinity when the normal angles of highly oblique waves approach the resonance cone angle, we set the pitch angle diffusion coefficient to zero when the refraction index is larger than 200 (Ma et al., 2017). We assume that transmitter signals are present at L = 1.4-1.9 and are confined within the longitude range of [99˚, 129˚] and the latitude range of [-25˚, 25˚] following previous studies (Ma et al., 2017; Meredith et al., 2019; Liu et al., 2022). The background electron density is 1.5 times larger than the model proposed by Ozhogin et al. (2012). Higher

electron densities lead to lower resonant energies. In contrast, higher magnetic fields produce higher resonant energies.

The pitch angle diffusion coefficients used in the simulation at six given L values are shown in Figure 4. The scattering effects from different resonance orders are evident in each panel. The resonance energy of electrons increases with resonance order N=1, N=-1, and N=2, respectively. At L=1.4, the pitch angle diffusion coefficients from N=-1, and N=2 are non-zero only for electrons with energies higher than 700 keV (Figure 4a). Thus, their effects are not shown. In addition, the diffusion coefficients from N=1 are mainly in the drift loss cone (indicated by the white dashed lines), suggesting that few electrons can be scattered from the trapped population into the drift loss cone at L=1.4. Accordingly, there is no wisp signature in simulation results at L=1.4 (Figure 4b-f). The resonant energy of electrons from different orders decreases at higher L, leading to lower energies of wisps at higher L (Figure 4b-f). Diffusion coefficients from N=1 and N=2 are stronger than those from N=-1. As a result, the first and third wisps are stronger than the second wisp. We assume unducted VLF waves (center wave normal angle is 65°) in the simulation, which is consistent with previous ray-tracing calculations during quiet geomagnetic conditions (Koons et al., 1981; Able & Thorne, 1998). Statistical studies also suggest that unducted signals have a dominant occurrence in the inner belt and ducted signals show a higher occurrence rate during/after magnetic active periods (Singh, 1976; Gu et al., 2021).

4. Discussion and Conclusions

Our analysis of multiple wisps measured by CIRBE/REPTile-2 demonstrates that these observations are produced by harmonic cyclotron resonances of VLF transmitter signals from the NWC station with energetic electrons. This is the first observational evidence of multiple

harmonic resonances occurring simultaneously in space. Due to the narrow bandwidth of the NWC transmitter signals and the high energy resolution measurements of REPTile-2, scattering from different resonance orders is clearly distinguished in both observations and simulations. The pitch-angle diffusion coefficients (Figure 4) used in the simulation are calculated based on quasi-linear theory. The consistency between observations and simulations validates quasi-linear theory in quantifying the scattering effects of whistler mode waves. Although quasi-linear theory has been established for ~60 years in radiation belt physics (Kennel & Engelmann, 1966; Lerche, 1968; Lyons et al., 1971; Lyons, 1974; Summers et al., 2007) and has successfully explained many observational phenomena (Thorne et al., 2013; Horne et al., 2008; Xiao et al., 2015; Shprits et al., 2016; Zhao et al., 2019b), the exact values of diffusion coefficients are still under debate due to the complex calculation process and the numerous assumptions (Cunningham, 2023). Multiple wisps provide an excellent opportunity to examine the theoretical calculation of diffusion coefficients by different resonance orders. Wisps can also be used to infer wave normal angles and amplitudes of NWC transmitter signals in the inner belt. Previous studies suggest that unducted VLF transmitter signals mainly occur at $L<1.5$ and have weak scattering effects on energetic electrons (Gamble et al., 2008; Clilverd et al., 2008). Multiple wisps demonstrate that highly oblique transmitter signals certainly exist at L=1.4-1.9 and produce significant scattering effects on energetic electrons. Parallel transmitter signals have no scattering effects at higher resonance orders (N=-1,2). Figure S1-S2 show simulation results of wisps produced by transmitter signals with 40° and 15° center wave normal angle. Two wisps occur in Figure S1 and only one wisp is clearly observed in Figure S2, demonstrating the requirement of highly oblique transmitter signals in the formation of multiple wisps. The amplitude of transmitter signals that leak into higher altitudes is also highly debated, ranging from a few pT to hundreds

of pT (Koons et al., 1981; Ma et al., 2017; Shen et al., 2022). The multiple wisps on 28-29 August 2023 suggest that NWC transmitter signals in the inner belt are around 20 pT, which is comparable to previous inferred transmitter signal amplitudes (~15 pT) from the longitudinal distribution of quasi-trapped electron fluxes scattered by NWC transmitter signals (Liu et al., 2022).

In theory, signals with different frequencies from three distinct VLF transmitter stations can also produce multiple wisps with only the N=1 cyclotron resonance. When the wisps in Figure 2 were observed, together with the VLF transmitter station that is discussed above, NWC (19.8 kHz, 1000 kW, L=1.42), there are two more VLF transmitter stations on the nightside (See Figure S3): JJI (22.2 kHz, 100 kW, L=1.25) and unid25 (25 kHz, 250 kW, L=1.32). However, we note that the transmitted power of the JJI and unid25 stations is much weaker (Meredith et al., 2019). In addition, their frequencies are higher than the NWC station, suggesting that the energy of electrons scattered by these two stations would be lower (Summers et al., 2007). Instead, we observe that the second and third wisps in **Fig. 2** have higher energies and weaker flux levels than the first wisp. Thus, we exclude the possibility that transmitter signals from different stations can produce the multiple wisps reported in this study.

Scattering by high-power VLF waves has been considered a promising method for controlling the radiation belts (Sauvaud et al., 2008; Carlsten et al., 2019; Foster et al., 2016; Ni et al., 2022). With harmonic cyclotron resonances, electrons scattered by human-made VLF waves expand to higher energies, leading to a stronger loss of energetic electrons in Earth's inner belt. Our results shed new light on developing artificial radiation belt remediation techniques to mitigate radiation hazards on spacecraft and astronauts.

Acknowledgments

We thank the entire CIRBE team. Especially, we owe our deepest gratitude to Rick Kohnert of LASP, who was the project manager for CIRBE and guided engineers and students since the “cradle” phase of CIRBE. Rick passed away before the launch of CIRBE, but we are certain that he would be proud of the contributions that CIRBE has made and will continue to make to the scientific community. This work was supported by the NASA Grants 80NSSC19K0995 and 80NSSC21K0583, and NSF Grant AGS 1834971.

Conflict of Interest

Authors declare that they have no competing interests.

Data Availability Statement

The CIRBE/REPTile-2 data are publicly available at https://doi.org/10.6084/m9.figshare.25751286.

References

Abel, B., & Thorne, R. M. (1998). Electron scattering loss in Earth's inner magnetosphere: 1. Dominant physical processes. *Journal of Geophysical Research*, 103, 2385–2396. https://doi.org/10.1029/97JA02919

Albert, J. M. (2007). Simple approximations of quasi-linear diffusion coefficients. *Journal of Geophysical Research*, 112, A12202. https://doi.org/10.1029/2007JA012551

Baker, D. N., Kanekal, S. G., Hoxie, V. C., Henderson, M. G., Li, X., Spence, H. E., et al. (2013). A long-lived relativistic electron storage ring embedded within the Earth’s outer Van

Allen radiation zone. *Science*, 340(6129), 186–190. https://doi.org/10.1126/science.1233518

Baker, D. N., An, J., Hoxie, V. C., Thorne, R. M., Foster, J. C., Li, X., et al. (2014). An impenetrable barrier to ultrarelativistic electrons in the Van Allen radiation belts. *Nature*, 515, 7528. https://doi.org/10.1038/nature13956

Bortnik, J., Thorne, R. M., & Meredith, N. P. (2008). The unexpected origin of plasmaspheric hiss from discrete chorus emissions. *Nature*, 452, 62–66. https://doi.org/10.1038/nature06741

Carlsten, B. E., Colestock, P. L., Cunningham, G. S., Delzanno, G. L., & Shipman, K. A. (2019). Radiation-belt remediation using space-based antennas and electron beams. *IEEE Transactions on Plasma Science*, 47(5), 2045–2063. https://doi.org/10.1109/TPS.2019.2910829

Clilverd, M. A., Rodger, C. J., Gamble, R., Meredith, N. P., Parrot, M., Berthelier, J.-J., & Thomson, N. R. (2008). Ground-based transmitter signals observed from space: Ducted or nonducted? *Journal of Geophysical Research*, 113, A04211. https://doi.org/10.1029/2007JA012602

Cunningham, G. S. (2023). Resolution of a few problems in the application of quasilinear theory to calculating diffusion coefficients in heliophysics. *Journal of Geophysical Research: Space Physics*, 128, e2023JA031703. https://doi.org/10.1029/2023JA031703

Datlowe, D. W., & Imhof, W. L. (1990). Cyclotron resonance precipitation of energetic electrons from the inner magnetosphere. *Journal of Geophysical Research*, 95(A5), 6477–6491. https://doi.org/10.1029/JA095iA05p06477

Datlowe, D. (2006). Differences between transmitter precipitation peaks and storm injection peaks in low-altitude energetic electron spectra. *Journal of Geophysical Research*, 111, A12202. https://doi.org/10.1029/2006JA011957

Foster, J. C., Erickson, P. J., Baker, D. N., Jaynes, A. N., Mishin, E. V., Fennel, J. F., Li, X., Henderson, M. G., & Kanekal, S. G. (2016). Observations of the impenetrable barrier, the plasmapause, and the VLF bubble during the 17 March 2015 storm. *Journal of Geophysical Research: Space Physics*, 121, 5537–5548. https://doi.org/10.1002/2016JA022509

Gamble, R. J., Rodger, C. J., Clilverd, M. A., Sauvaud, J.-A., Thomson, N. R., Stewart, S. L., et al. (2008). Radiation belt electron precipitation by man-made VLF transmissions. *Journal of Geophysical Research*, 113, A10211. https://doi.org/10.1029/2008JA013369

Gu, W., Chen, L., Xia, Z., & Horne, R. B. (2021). Direct evidence reveals transmitter signal propagation in the magnetosphere. *Geophysical Research Letters*, 48, e2021GL093987. https://doi.org/10.1029/2021GL093987

Horne, R. B., Thorne, R. M., Glauert, S. A., Albert, J. M., Meredith, N. P., & Anderson, R. R. (2005). Timescale for radiation belt electron acceleration by whistler mode chorus waves. *Journal of Geophysical Research*, 110(A3). https://doi.org/10.1029/2004ja010811

Horne, R. B., Thorne, R. M., Glauert, S. A., Douglas Menietti, J., Shprits, Y. Y., & Gurnett, D. A. (2008). Gyro-resonant electron acceleration at Jupiter. *Nature Physics*, 4(4), 301–304. https://doi.org/10.1038/nphys897

Hua M., Li, W., Ni, B., Ma, Q., Green, A., Shen, X., et al. (2020). Very-low-frequency transmitters bifurcate energetic electron belt in near-Earth space. *Nature Communications*, 11(1), 4847. https://doi.org/10.1038/s41467-020-18545-y

Imhof, W. L., Gaines, E. E., & Reagan, J. B. (1973). Dynamic variations in intensity and energy spectra of electrons in the inner radiation belt. *Journal of Geophysical Research*, 78(22), 4568–4577. https://doi.org/10.1029/ja078i022p04568

Kasahara, S., Miyoshi, Y., Yokota, S., Mitani, T., Kasahara, Y., Matsuda, S., et al. (2018). Pulsating aurora from electron scattering by chorus waves. *Nature*, 554(7692), 337–340. https://doi.org/10.1038/nature25505

Kennel, C. F., & Engelmann, F. (1966). Velocity space diffusion from weak plasma turbulence in a magnetic field. *Physics of Fluids*, 9(12), 2377–2388. https://doi.org/10.1063/1.1761629

Khoo, L.-Y., Li, X., Selesnick, R. S., Schiller, Q., Zhang, K., Zhao, H., et al. (2022). On the challenges of measuring energetic particles in the inner belt: A Geant4 simulation of an energetic particle detector instrument, REPTile-2. *Journal of Geophysical Research: Space Physics*, 127(4), e2021JA030249. https://doi.org/10.1029/2021JA030249

Koons, H., Edgar, B., & Vampola, A. (1981). Precipitation of inner zone electrons by whistler mode waves from the VLF transmitters UMS and NWC. *Journal of Geophysical Research*, 86(A2), 640–648. https://doi.org/10.1029/JA086iA02p00640

Lerche, I. (1968). Quasilinear Theory of Resonant Diffusion in a Magneto-Active, Relativistic Plasma. *The Physics of Fluids*, 11(8), 1720–1727. https://doi.org/10.1063/1.1692186

Li, X., Selesnick, R., Schiller, Q., Zhang, K., Zhao, H., Baker, D. N., & Temerin, M. A. (2017). Measurement of electrons from albedo neutron decay and neutron density in near-Earth space. *Nature*, 552(7685), 382. https://doi.org/10.1038/nature24642–385.

Li, X., Kohnert, R., Palo, S., Selesnick, R., Khoo, L., & Schiller, Q. (2022). Two generations of CubeSat missions (CSSWE and CIRBE) to take on the challenges of measuring

relativistic electrons in Earth's magnetosphere. In 36th annual small satellite conference. Retrieved from https://digitalcommons.usu.edu/cgi/viewcontent.cgi?article=5311&context=smallsat

Li, X., Selesnick, R., Mei, Y., O'Brien, D., Hogan, B., Xiang, Z., et al. (2024b). First results from REPTile-2 measurements onboard CIRBE. *Geophysical Research Letters*, 51(3), e2023GL107521. https://doi.org/10.1029/2023GL107521

Li, X. (2024a). Unveiling energetic particle dynamics in the near-Earth environment from CubeSat missions. *AGU Advances,* 5, e2024AV001256. https://doi.org/10.1029/2024AV001256

Liu, Y., Xiang, Z., Ni, B., Li, X., Zhang, K., Fu, S., et al. (2022). Quasi-trapped electron fluxes induced by NWC transmitter and CRAND: Observations and simulations. *Geophysical Research Letters*, 49(5), e2021GL097443. https://doi.org/10.1029/2021GL097443

Lyons, L. R., Thorne, R. M., & Kennel, C. F. (1971). Electron pitch-angle diffusion driven by oblique whistler-mode turbulence. *Plasma Physics*, 6, part 3, 589–606.

Lyons, L. R. (1974). Pitch angle and energy diffusion coefficients from resonant interactions with ion-cyclotron and whistler waves. *Journal of Plasma Physics*, 12, 417–432.

Ma, Q., Mourenas, D., Li, W., Artemyev, A., & Thorne, R. M. (2017). VLF waves from ground-based transmitters observed by the Van Allen Probes: Statistical model and effects on plasmaspheric electrons. *Geophysical Research Letters*, 44, 6483–6491. https://doi.org/10.1002/2017GL073885

Mann, I. R., Ozeke, L. G., Murphy, K. R., Claudepierre, S. G., Turner, D. L., Baker, D. N., Rae, I. J., Kale, A., Milling, D. K., Boyd, A. J., Spence, H. E., Reeves, G. D., Singer, H. J., Dimitrakoudis, S., Daglis, I. A., & Honary, F. (2016). Explaining the dynamics of the

ultra-relativistic third Van Allen radiation belt. *Nature Physics*, 12(10), 978–983. https://doi.org/10.1038/nphys3799

Meredith, N. P., Horne, R. B., Clilverd, M. A., Horsfall, D., Thorne, R. M., & Anderson, R. R. (2006). Origins of plasmaspheric hiss. *Journal of Geophysical Research*, 111, A09217. https://doi.org/10.1029/2006JA011707

Meredith, N. P., Horne, R. B., Clilverd, M. A., & Ross, J. P. J. (2019). An investigation of VLF transmitter wave power in the inner radiation belt and slot region. *Journal of Geophysical Research: Space Physics*, 124, 5246–5259. https://doi.org/10.1029/2019JA026715

Mihalov, J. D., & White, R. S. (1966). Energetic electron spectra in the radiation belts. *Journal of Geophysical Research*, 71(9), 2217-2226. https://doi.org/10.1029/JZ071i009p02217

Ni, B., Thorne, R. M., Shprits, Y. Y., & Bortnik, J. (2008). Resonant scattering of plasma sheet electrons by whistler-mode chorus: Contribution to diffuse auroral precipitation. *Geophysical Research Letters*, 35, L11106. https://doi.org/10.1029/2008GL034032

Ni, B., Thorne, R. M., Shprits, Y. Y., Orlova, K. G., & Meredith, N. P. (2011). Chorus-driven resonant scattering of diffuse auroral electrons in nondipolar magnetic fields. *Journal of Geophysical Research: Space Physics*, 116, 6225. https://doi.org/10.1029/2011JA016453

Ni, B., Hua, M., Gu, X., Fu, S., Xiang, Z., Cao, X., & Ma, X. (2022). Artificial modification of Earth's radiation belts by ground-based very-low-frequency (VLF) transmitters. *Science China Earth Sciences*, 65(3), 391–413. https://doi.org/10.1007/s11430-021-9850-7

Omura, Y., Katoh, Y., & Summers, D. (2008). Theory and simulation of the generation of whistler-mode chorus. *Journal of Geophysical Research*, 113, A04223. https://doi.org/10.1029/2007JA012622

Ozhogin, P., Tu, J., Song, P., & Reinisch, B. W. (2012). Field-aligned distribution of the plasmaspheric electron density: An empirical model derived from the IMAGE RPI measurements. *Journal of Geophysical Research*, 117, A06225. https://doi.org/10.1029/2011JA017330

Roth, I., Temerin, M., & Hudson, M. (1999). Resonant enhancement of relativistic electron fluxes during geomagnetically active periods. *Annales Geophysicae*, 17, 631–638.

Sauvaud, J. A., Maggiolo, R., Jacquey, C., Parrot, M., Berthelier, J. J., Gamble, R. J., & Rodger, C. J. (2008). Radiation belt electron precipitation due to VLF transmitters: Satellite observations. *Geophysical Research Letters*, 35, L09101. https://doi.org/10.1029/2008GL033194

Selesnick, R., Blake, J., & Mewaldt, R. (2003). Atmospheric losses of radiation belt electrons. *Journal of Geophysical Research: Space Physics*, 108(A12), e2003JA010160. https://doi.org/10.1029/2003ja010160

Selesnick, R. S., Albert, J. M., & Starks, M. J. (2013). Influence of a ground-based VLF radio transmitter on the inner electron radiation belt. *Journal of Geophysical Research: Space Physics*, 118, 628–635. https://doi.org/10.1002/jgra.50095

Selesnick, R. S. (2015). Measurement of inner radiation belt electrons with kinetic energy above 1 MeV. *Journal of Geophysical Research: Space Physics*, 120(10), 8339–8349. https://doi.org/10.1002/2015JA021387

Selesnick, R. S. (2015). High-energy radiation belt electrons from CRAND. *Journal of Geophysical Research: Space Physics*, 120, 2912–2917. https://doi.org/10.1002/2014JA020963

Shen, Y., Artemyev, A. V., Ma, Q., Zhang, X.-J., Mourenas, D., Tsai, E., et al. (2022). Inner belt wisp precipitation measured by ELFIN: Regimes of energetic electron scattering by VLF transmitter waves. *Journal of Geophysical Research: Space Physics*, 127, e2022JA030968. https://doi.org/10.1029/2022JA030968

Shprits, Y. Y., Drozdov, A. Y., Spasojevic, M., Kellerman, A. C., Usanova, M. E., Engebretson, M. J., Agapitov, O. V., Zhelavskaya, I. S., Raita, T. J., Spence, H. E., Baker, D. N., Zhu, H., & Aseev, N. A. (2016a). Wave-induced loss ofultra-relativistic electrons in the Van Allen radiation belts. *Nature Communications*, 7(1), 883. https://doi.org/10.1038/ncomms12883

Singh, B. (1976), On the ground observation of whistlers at low latitudes, *Journal of Geophysical Research*, 81(13), 2429–2432, https://doi.org/10.1029/JA081i013p02429

Summers, D., Ni, B., & Meredith, N. P. (2007). Timescales for radiation belt electron acceleration and loss due to resonant wave-particle interactions: 1. Theory. *Journal of Geophysical Research*, 112, A04206. https://doi.org/10.1029/2006JA011801

Teng, S., Wu, Y., Harada, Y., Bortnik, J., Zonca, F., Chen, L., & Tao, X. (2023). Whistler-mode chorus waves at Mars. *Nature Communications*, 14(1), 3142. https://doi.org/10.1038/s41467-023-38776-z

Thorne, R. M., Ni, B., Tao, X., Horne, R. B., & Meredith, N. P. (2010). Scattering by chorus waves as the dominant cause of the Earth's diffuse aurora. *Nature*, 467, 943–946. https://doi.org/10.1038/nature09467

Thorne, R. M., Li, W., Ni, B., Ma, Q., Bortnik, J., Chen, L., Baker, D. N., Spence, H. E., Reeves, G. D., Henderson, M. G., Kletzing, C. A., Kurth, W. S., Hospodarsky, G. B., Blake, J. B., Fennell, J. F., Claudepierre, S. G., & Kanekal, S. G. (2013a). Rapid local acceleration of

relativistic radiation-belt electrons by magnetospheric chorus. *Nature*, 504(7480), 411–414. https://doi.org/10.1038/nature12889

Tu, W., Selesnick, R., Li, X., & Looper, M. (2010). Quantification of the precipitation loss of radiation belt electrons observed by SAMPEX. *Journal of Geophysical Research*, 115, A07210. https://doi.org/10.1029/2009JA014949

Vampola, A. L., & Kuck, G. A. (1978). Induced precipitation of inner zone electrons: 1. Observations. *Journal of Geophysical Research*, 83(A6), 2543–2551. https://doi.org/10.1029/JA083iA06p02543

Xiang, Z., Li, X., Selesnick, R., Temerin, M. A., Ni, B., Zhao, H., et al. (2019). Modeling the quasi-trapped electron fluxes from cosmic ray albedo neutron decay (CRAND). *Geophysical Research Letters*, 46(4), 1919–1928. https://doi.org/10.1029/2018GL081730

Xiang, Z., Li, X., Temerin, M. A., Ni, B., Zhao, H., Zhang, K., & Khoo, L. Y. (2020a). On energetic electron dynamics during geomagnetic quiet times in Earth's inner radiation belt due to atmospheric collisional loss and cosmic ray albedo neutron decay (CRAND) as a source. *Journal of Geophysical Research: Space Physics*, 125, e2019JA027678. https://doi.org/10.1029/2019JA027678

Xiang, Z., Li, X., Ni, B., Temerin, M. A., Zhao, H., Zhang, K., & Khoo, L. Y. (2020b). Dynamics of energetic electrons in the slot region during geomagnetically quiet times: Losses due to wave particle interactions versus a source from cosmic ray albedo neutron decay (CRAND). *Journal of Geophysical Research: Space Physics*, 125(9), e2020JA028042. https://doi.org/10.1029/2020JA028042

Xiao, F., Yang, C., Su, Z., Zhou, Q., He, Z., He, Y., Baker, D. N., Spence, H. E., Funsten, H. O., & Blake, J. B. (2015). Wave-driven butterfly distribution of Van Allen belt relativistic electrons. *Nature Communications*, 6, 8590. https://doi.org/10.1038/ncomms9590

Zhang, K., Li, X., Zhao, H., Schiller, Q., Khoo, L.-Y., Xiang, Z., et al. (2019). Cosmic Ray Albedo Neutron Decay (CRAND) as a source of inner belt electrons: Energy spectrum study. *Geophysical Research Letters*, 46, 544–552. https://doi.org/10.1029/2018GL080887

Zhao, H., Johnston, W. R., Baker, D. N., Li, X., Ni, B., Jaynes, A. N., et al. (2019a). Characterization and evolution of radiation belt electron energy spectra based on the Van Allen Probes measurements. *Journal of Geophysical Research: Space Physics*, 124, 4217–4232. https://doi.org/10.1029/2019JA026697

Zhao, H., Ni, B., Li, X., Baker, D. N., Johnston, W. R., Zhang, W., et al. (2019b). Plasmaspheric hiss waves generate a reversed energy spectrum of radiation belt electrons. *Nature Physics*, 15(4), 367–372. https://doi.org/10.1038/s41567-018-0391-6

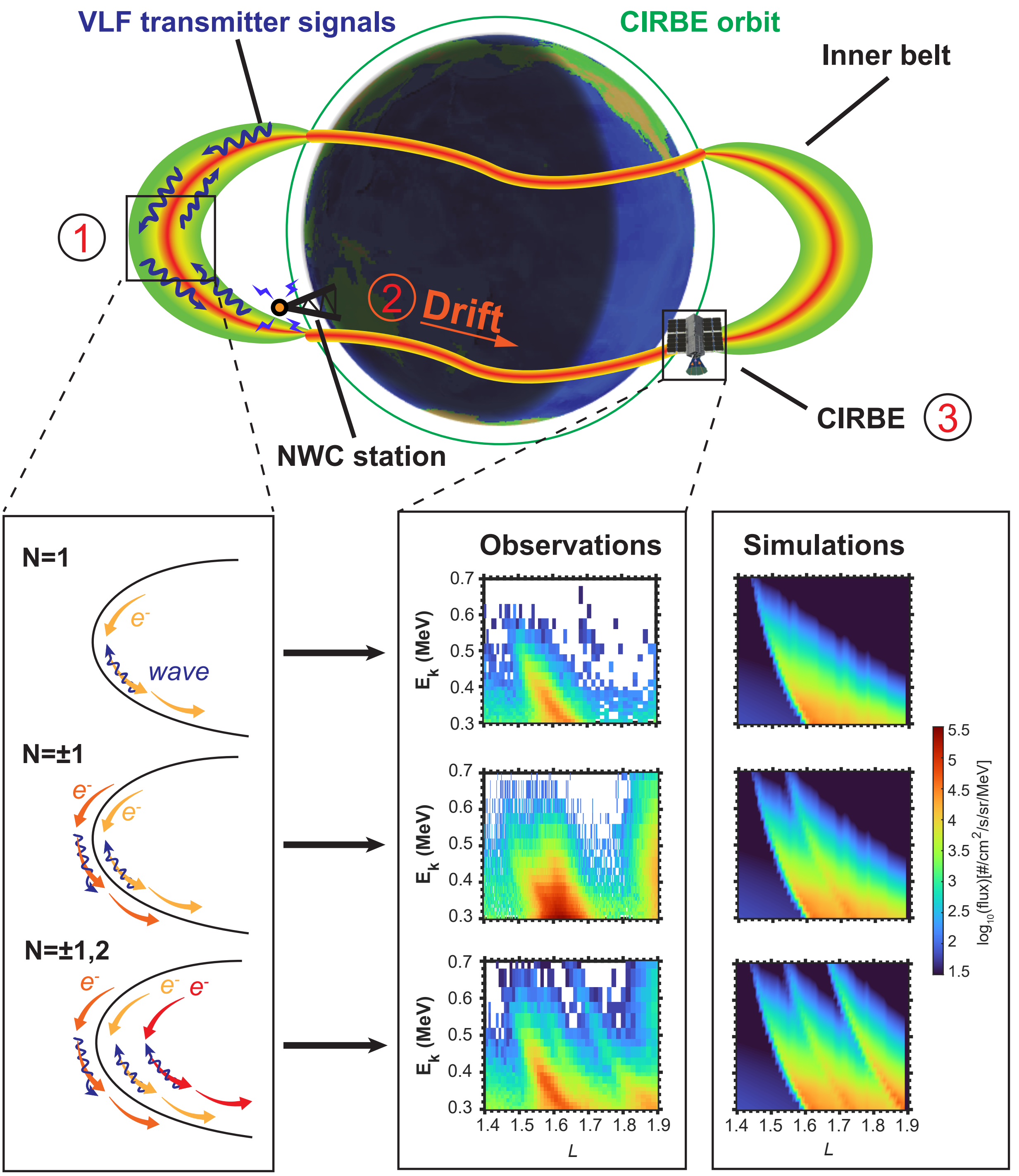

VLF transmitter signals
CIRBE orbit
Inner belt
1
2 Drift
NWC station
CIRBE 3
N=1
e-
wave
N=±1
e-
e-
N=±1,2
e-
e-
e-
Observations
Simulations
E_k (MeV)
0.7
0.6
0.5
0.4
0.3
1.4 1.5 1.6 1.7 1.8 1.9
L
log_{10}(flux)[#/cm^2/s/sr/MeV]
5.5
5
4.5
4
3.5
3
2.5
2
1.5

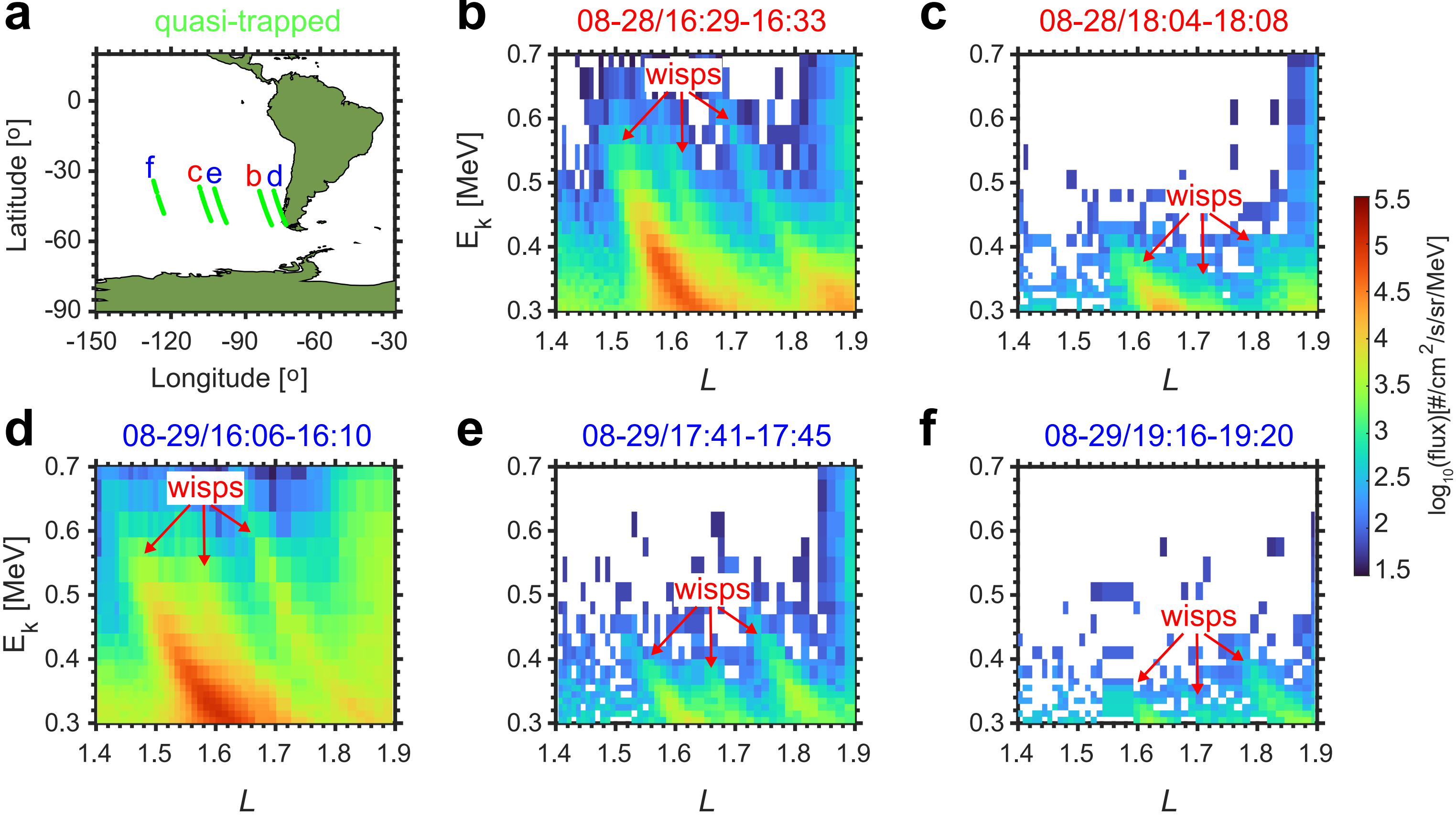

a
quasi-trapped
Latitude [o]
Longitude [o]
f
c
e
b
d
b
08-28/16:29-16:33
c
08-28/18:04-18:08
d
08-29/16:06-16:10
e
08-29/17:41-17:45
f
08-29/19:16-19:20
wisps
E$_k$ [MeV]
L
log$_{10}$(flux)[#/cm^2/s/sr/MeV]

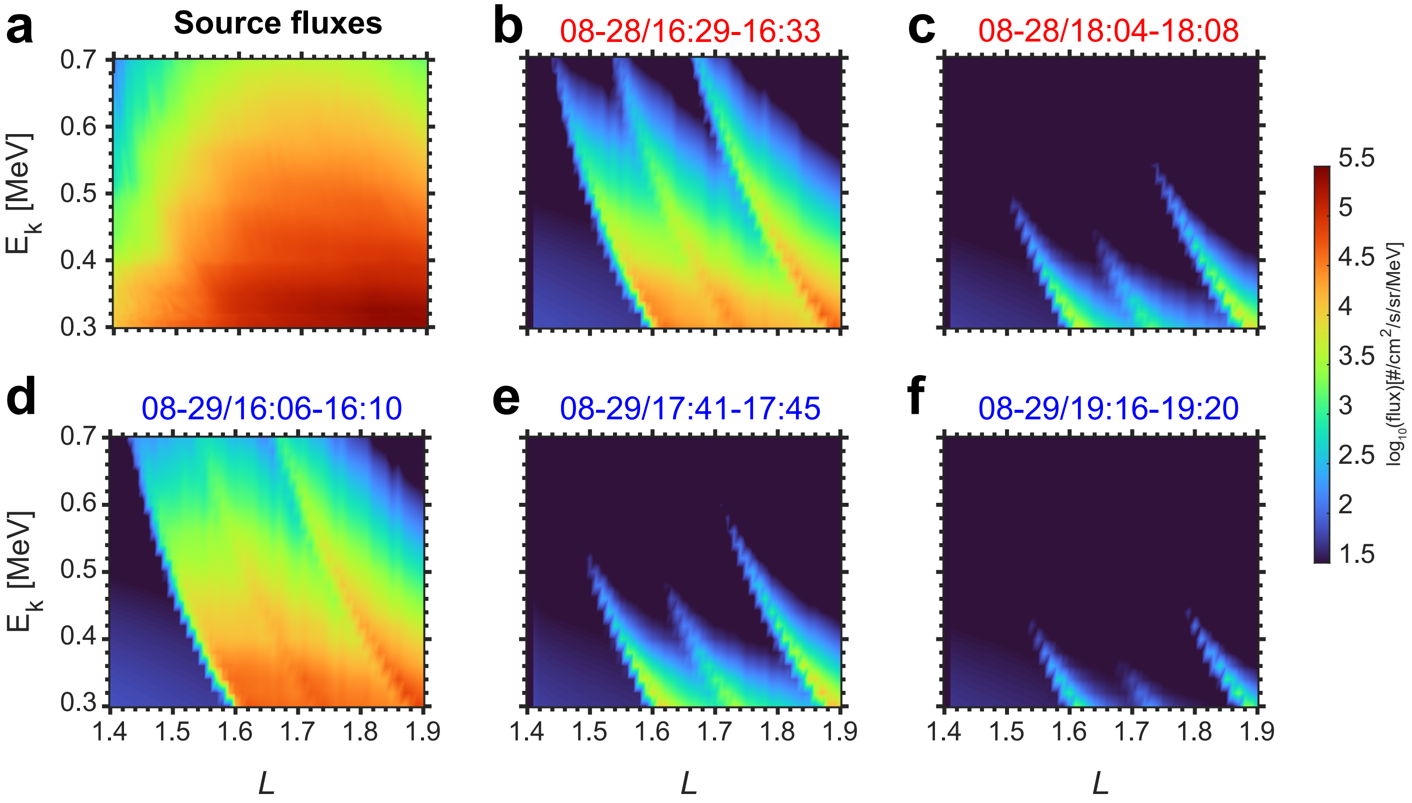

a
Source fluxes
b
08-28/16:29-16:33
c
08-28/18:04-18:08
d
08-29/16:06-16:10
e
08-29/17:41-17:45
f
08-29/19:16-19:20
0.7
0.6
0.5
0.4
0.3
E_k [MeV]
1.4
1.5
1.6
1.7
1.8
1.9
L
5.5
5
4.5
4
3.5
3
2.5
2
1.5
$\log_{10}$(flux)[#/cm^2/s/sr/MeV]

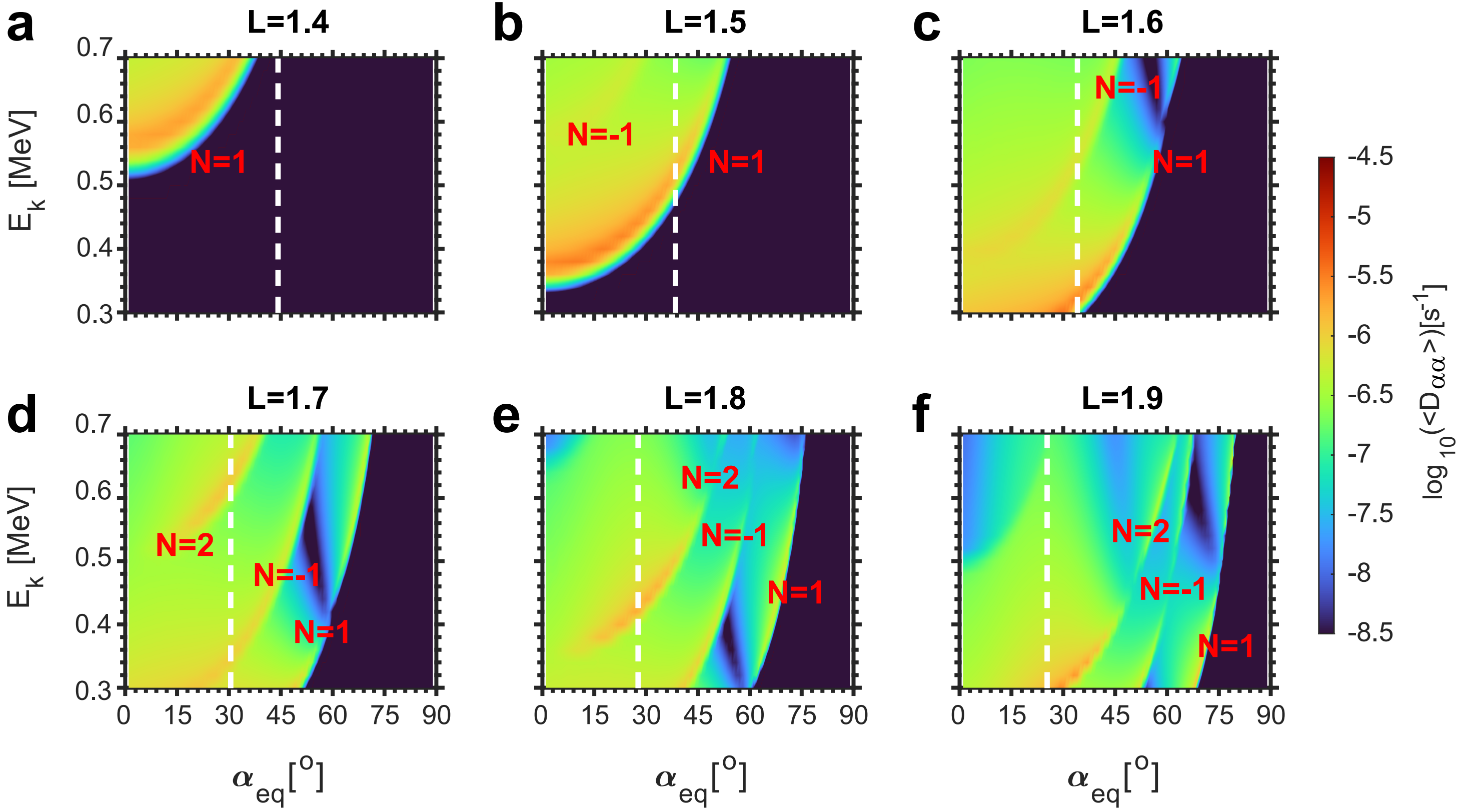

a
L=1.4
N=1
b
L=1.5
N=-1
N=1
c
L=1.6
N=-1
N=1
d
L=1.7
N=2
N=-1
N=1
e
L=1.8
N=2
N=-1
N=1
f
L=1.9
N=2
N=-1
N=1
E_k [MeV]
0.3
0.4
0.5
0.6
0.7
0
15
30
45
60
75
90
α_{eq}[°]
$\log_{10}(<D_{\alpha\alpha}>)[s^{-1}]$
-4.5
-5
-5.5
-6
-6.5
-7
-7.5
-8
-8.5
