# Supplementary material for: Earth‐Based Transmitters Trigger Precipitation of Inner Radiation Belt Electrons: Unveiling Observations and Modeling Results
Source: AGU Adv. 2024 Nov 21;5(6):e2024AV001354. doi: 10.1029/2024AV001354 (PMC11600777; doi:10.1029/2024AV001354)
Supplement: Supplementary file 4 — Author Response to Peer Review Comments [file AGA2-5-0-s005.pdf]

We thank the reviewer for careful reading of the manuscript and the valuable comments for improvements. Accordingly, we have modified the text and presented more discussions. All the modifications and changes are shown in the revised manuscript as tracked changes. Our responses to the reviewers' comments are listed below.

Responses to Reviewer #1:

This is a very interesting paper presenting an observational evidence of the scattering by VLF transmitters and even demonstrating distinctive features of different harmonic scattering. The paper is well written and I only have minor comments.

Comments:

1. The oblique VLF transmitter scattering has been done in the past including resonances from -5 to +5 in Kim, et al. (2011),

Reply:

We thank the reviewer for pointing it out. We have cited Kim et al., (2011) in Lines 263 and 340.

2. I would also suggest to discuss that previous estimates may have underestimated the transmitter scattering as they used a correction according to Starks 2008
Starks, M. J., R. A. Quinn, G. P. Ginet, J. M. Albert, G. S. Sales, B. W. Reinisch, and P. Song (2008), Illumination of the plasmasphere by terrestrial very low frequency transmitters: Model validation, J. Geophys. Res., 113, A09320, doi:10.1029/2008JA013112.
That estimate may have been underestimation as there is a broad illuminated area and the peak intensity may be missed by a spacecraft.
I would review more carefully the previous work and also discuss the estimates of Abel and Thorne and Startks et al

Reply:

We thank the reviewer for these valuable suggestions. We have discussed the previous estimates of wave amplitudes of VLF transmitter signals and their scattering effects in Lines 333-350 as:

"Abel and Thorne (1998) combined eight most powerful VLF transmitters at the time into two frequency groups (17.1 and 22.3 kHz) and calculated their amplitudes based on the method of Inan et al. (1984). They suggested that the amplitudes of the 22.3 kHz signal group are 15-20 pT at L=1.5-2, comparable to those inferred from multiple wisps. Note that the NWC transmitter was operating at 22.3 kHz at the time and had changed to 19.8 kHz when multiple wisps were observed by CIRBE/REPTile-2. The electron lifetime calculated in Abel and Thorne (1998) suggests that transmitter signals significantly influence energetic electron distributions at L<2.8. However, Kim et al. (2011) found that transmitter signals do not contribute to the loss of relativistic electrons in the slot region using a much smaller wave amplitude (0.8 pT) in the 3D Versatile Electron Radiation Belt (VERB) code. The smaller wave

amplitude is estimated based on Starks et al. (2008), which may underestimate the transmitter intensity due to a broad illuminated area, and the peak intensity may be missed by spacecraft. In an updated study, Starks et al. (2020) obtained larger wave amplitudes of NWC transmitter signals (a few pT with longitude-averaged) based on first-principles modeling of VLF transmitter from the source to the plasmasphere. It should be noted that the 20 pT amplitude of NWC transmitter signals inferred from multiple wisps are local wave intensities, namely not longitude averaged. To study long-term scattering effects induced by transmitter signals, the longitude dependence of signal intensities should be considered."

3. For the references to FDC I would suggest to cite the study of
Shprits, Y. Y., and B. Ni (2009), Dependence of the quasi-linear scattering rates on the wave normal distribution of chorus waves, J. Geophys. Res., 114, A11205, doi:10.1029/2009JA014223.
Which was the first time the code was used for the radiation belts. That would also give a proper credit to both of the developers of the FDC code.

Reply:

We thank the reviewer for pointing this out. We have cited Shprits and Ni (2009) in Line 251.

Finally, we would like to thank the reviewer again for positive, valuable and constructive comments and suggestions, which help a lot to improve the quality of our paper. We hope that the reviewer will be satisfied with our responses and revisions, and we look forward to hearing from the reviewer soon.
