# Supplementary material for: Earth‐Based Transmitters Trigger Precipitation of Inner Radiation Belt Electrons: Unveiling Observations and Modeling Results
Source: AGU Adv. 2024 Nov 21;5(6):e2024AV001354. doi: 10.1029/2024AV001354 (PMC11600777; doi:10.1029/2024AV001354)
Supplement: Supplementary file 5 — Author Response to Peer Review Comments [file AGA2-5-0-s003.pdf]

We thank the reviewer for careful reading of the manuscript and valuable comments/suggestions for improvements. Accordingly, we have modified the text and presented more details and discussions. All the modifications and changes are shown in the revised manuscript as tracked changes. Our responses to the reviewer's comments/suggestions are listed below. The line numbers in the responses correspond to those in the change-tracked file.

**Responses to Reviewer #2:**

*The article "Earth-Based Transmitters Trigger Precipitation of Inner Radiation Belt Electrons: Unveiling Observations and Modeling Results" written by Xiang et al. and submitted to JGR Advances is about observations of electrons flux at LEO which present a typical "wisp" shape. Observations are made from CIRBE, a cube-sat built and managed by the authors' team. This wisp has been observed in the past, first by Sauvaud et al. in 2008 from DEMETER. What is particularly interesting and important in CIRBE's results is the observation of multiple wisps. These wisps have been attributed to the NWC VLF transmitter signals emitted from Australia in the past. Most of the article is focused on the authors' attempt to explain the flux by modeling and simulation, with explanations that I question in following.*

*The observations of CIRBE of the wisp are very interesting, in particular their shape with multiple wisps and the variability among them. With proper discussion and comparisons to literature, this would be sufficient to make an interesting article, may be not in JGR Advances.*

*The main concerns I have are for the explanations given by the authors. The authors develop ideas based on which "harmonics" during wave-particle interactions would dominate and control the interaction. They argue that different resonance orders have never been examined by observations, claiming they can differentiate between harmonics. This can be questioned as the summation over all harmonics at a given location and energy should make this not possible, or hardly possible, likely not observable. I find their demonstration partially supported in this article. Questions are based on the fact that all harmonics contribute at a given energy and location and there is most of the time the domination of the n=-1 harmonic over all the others. Exceptions matter though, but would have to be proven. Generally, if conditions were such that a lower harmonic dominated the n=-1 harmonics, this would have to occur on a limited pitch angle range. There would be conditions, which would have to be discussed in the article. This would also be possible or favored with large wave normal angle. But high wave normal angle will in turn drastically reduce the scattering rate associated to wave particle interactions. As all harmonics shall contribute, Figure 1 sketch is doubtful: if the bottom panel is the results of all contributing harmonics (|n|<3), which we could believe, this would lead to three wisps occurring at 3 different L-shells (bottom panel) according to the authors, but how would the one single wisp configuration (top panel) possibly exist? Such*

*configuration would be dominated by only one harmonic. But why would the other two wisps not exist since they were just proven to exist by the observation in the bottom panel?*

*Such discussion is absent of this article and lacking and conclusions are doubtful and drawn too quickly. Moreover, some part of this discussion has been published in Cunningham et al. GRL 2021, where these authors observe for the first time at LEO high flux of electrons above 500 keV, which they proved to be related to the NWC (by comparing on and off periods). These authors evaluate the resonance condition for the two first main harmonics (see their figure 2) and can this way relate flux, energy and location, while discussing the harmonics importance. In their discussion, they show how the harmonic number can influence the location, similarly to what is done here. There is some anteriority of this results. The Cunningham et al. 2021 GRL article is presently not cited by the authors, but cited in their previous articles (Liu et al., 2022; Li et al., 2024).*

*I list in following the major questions/comments which I recommend the authors perform to assess their claim before publication.*

*1. The authors should demonstrate that, although harmonics accumulate at the same energy/location, they can be differentiated. That will require a discussion on the observed and computed pitch angle range (and wave normal). The author can choose a relevant energy at which they should plot the diffusion coefficients versus pitch angle for the following harmonics n=-3 …n=0… n=3 as well as all harmonics summed, all plotted on the same plot. That will allow to show the dominance of one harmonic versus the others and the overall contribution of each within the sum, for a given pitch angle range. This should be done at relevant L-shell of the study, each indicative of a different wisp. I recommend a fixed energy of E=(300, 500) keV and fixed L-shell L=(1.5, 1.7, 1.9) with Daa plotted versus pitch angle in 1D. This makes a figure with 6 panels, which can be put in the supplementary material.*

**Reply:**

The pitch angle diffusion coefficients of 300 keV and 500 keV electrons from different resonance orders (n= -3 to n=3) at L=1.5, 1.6, and 1.7 are shown below:

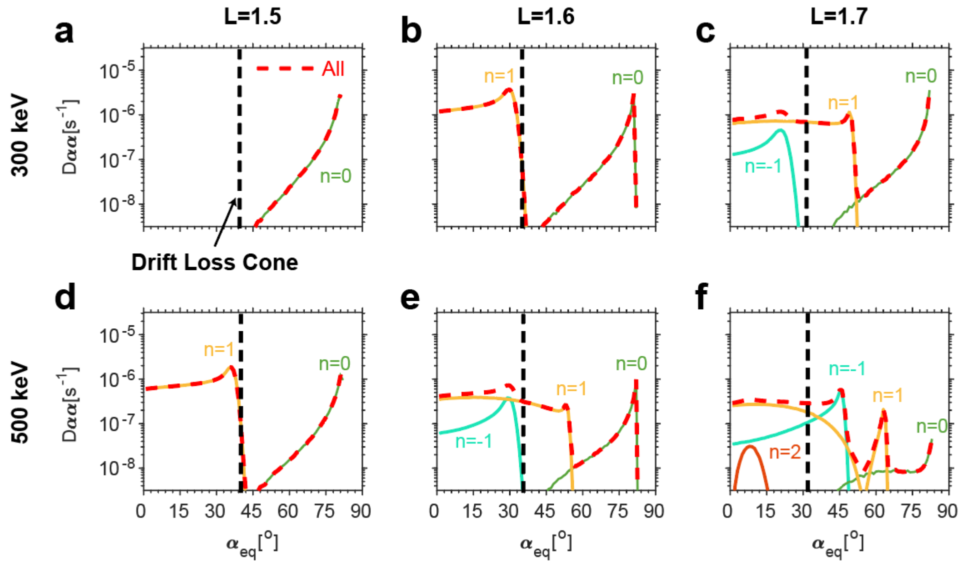


Figure R1. Pitch angle diffusion coefficients for NWC transmitter signals with 20 pT wave amplitudes and 65° centered wave normal angle. Pitch angle diffusion coefficients from different resonance orders are indicated by solid curves with different colors. The summations of difference resonance orders are indicated by the red dashed curves. The vertical black dashed lines represent drift loss cones at the selected L values. There is no diffusion coefficient from n=-3, -2, and 3 since the corresponding resonance energies are much higher than 500 keV.

The multiple wisps observed by CIRBE/REPTile-2 are quasi-trapped electrons (with pitch angles larger than the local bounce loss cone but smaller than the drift loss cone). These electrons loss into the SAA within one drift period. Therefore, pitch angle diffusion coefficients close to the drift loss cones significantly determine the flux levels of the wisps. For 300 keV electrons, there is no scattering close to drift loss cone (Figure R1a) at L=1.5 while n=1 dominant scattering at L=1.6 (Figure R1b) and 1.7 (Figure R1c). The importance of n= -1 cannot be seen in these L values. Thus, we replot Figure R1 at three L values (L=1.6, 1.75, 1.9) and show it below:

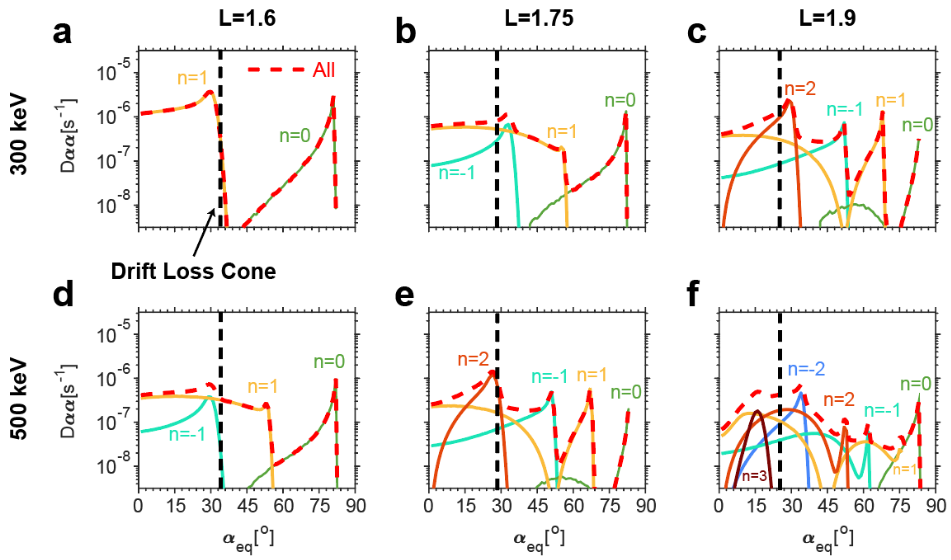


Figure R2. Same format as Figure R1 but for L=1.6, 1.75, and 1.9.

It can be observed that, for 300 keV electrons, n=1, -1, and 2 dominate the diffusion coefficients near drift loss cones at L=1.6, L=1.75, and L=1.9 (Figure R2a-R2c), respectively. Thus, three wisps are produced at L=1.6-1.9. Diffusion coefficients from n=1, 2 are stronger than n= -1. Accordingly, the flux level of second wisp is weaker than those of the first and third wisps. Those features are observed by CIRBE/REPTile-2 (Figure 2 in the main text) and simulated in the Drift-Diffusion-Source model (Figure 3 in the main text). These results demonstrate that harmonics can be differentiated. Figure R2 has been added in the supporting information as Figure S1.

*2. As explained above, increasing the wave normal angle will reduce the wave particle interactions. This effect has also a pitch angle dependence. A comparison between diffusion coefficient at large/small wave normal angle versus pitch angle at the above energy/location will allow a quantification of this effect. This makes another figure with 6 panels. The discussion in the text should indicate by how much the diffusion rate will be reduced by increasing the wave normal angle.*

**Reply:**

Pitch angle diffusion coefficients for NWC transmitter signals with 20 pT wave amplitudes and 15° centered wave normal angles (Figure R2 in the above responses is for 65° centered wave normal angles) are shown below:

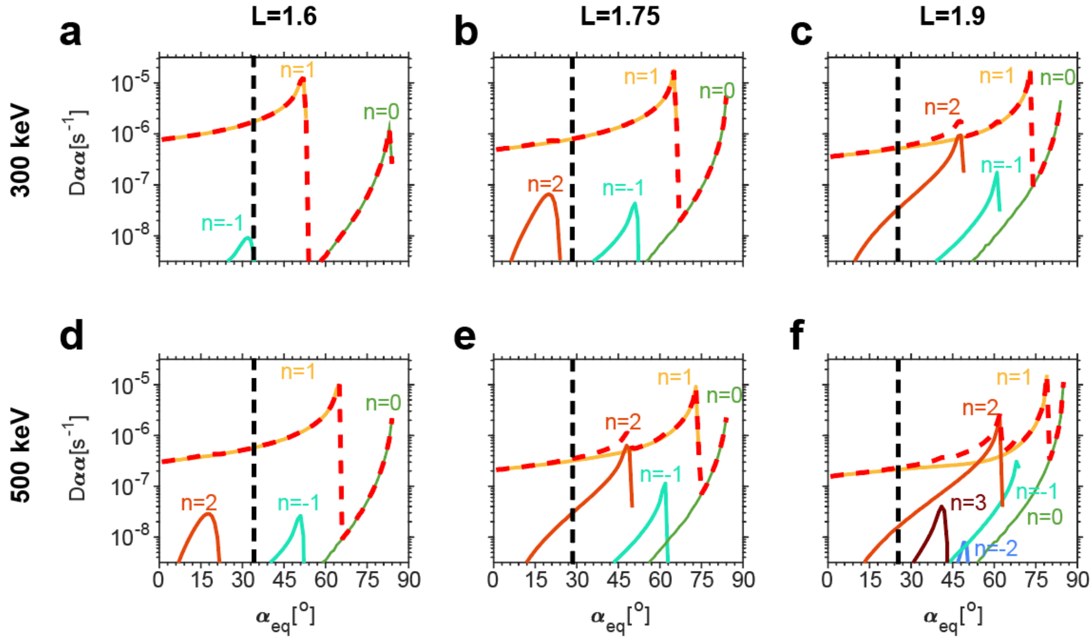


Figure R3. Same format as Figure R2 but for 15° centered wave normal angle

It can be observed that n=1 dominates the diffusion coefficient near the drift loss cone at the three selected L values. As a result, one wisp is generated in the Drift-Diffusion-Source model (see Figure S5 in the supporting information). This explains why sometimes one wisp was observed and sometimes multiple wisps were observed. This point has been added in Lines 143-144 to explain the observed single wisps in Figure 1.

The comparison between diffusion coefficients from small (15° centered wave normal

angle) and large (65 ° centered wave normal angle) wave normal angles are shown below:

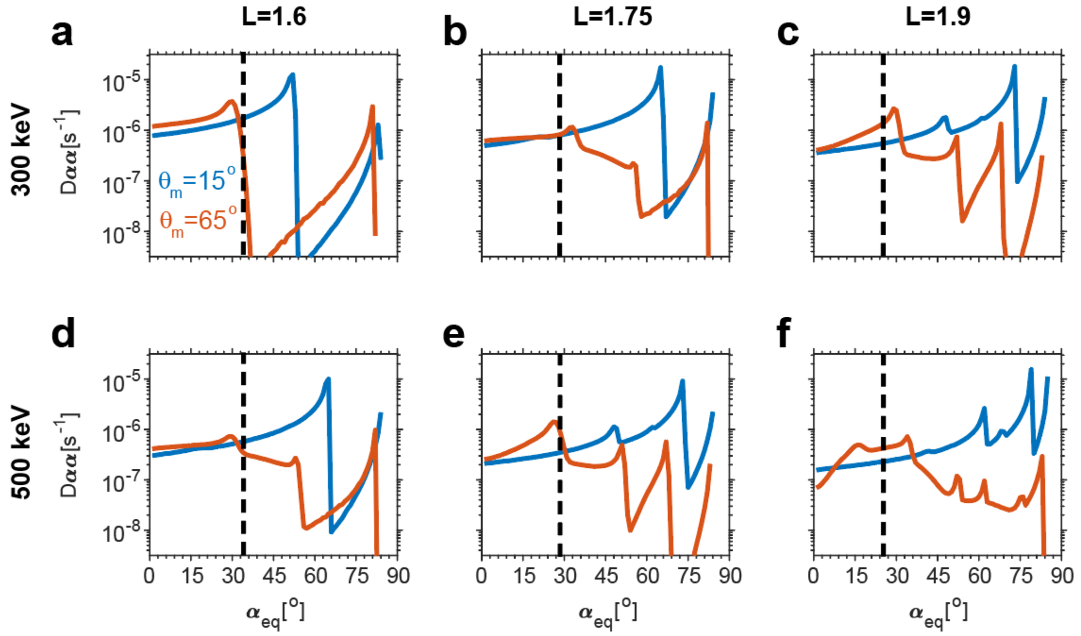


Figure R4. Comparison between pitch angle diffusion coefficients for small (15° centered wave normal angle) and large wave normal angle. Harmonics from n= -3 to n=3 are included.

Diffusion coefficients induced by transmitter signals with small wave normal angles are higher than those induced by highly oblique transmitter signals at larger pitch angles. However, close to the drift loss cones, the diffusion coefficients induced by oblique transmitter signals are larger, especially at higher L values (Figure R4c, R4e, R4f). Increasing the wave normal angles does not reduce the diffusion coefficients accounting for the formation of wisps. Thus, the multiple wisps generated by oblique transmitter signals have comparable/higher flux levels than the single wisps induced by parallel transmitter signals. Figure R3 and Figure R4 have been added in the supporting information as Figure S2, S3.

*3. The initial condition of the simulation is not plotted but should be.*

**Reply:**

The initial electron flux is the trapped electron flux measured by CIRBE/REPTile-2 at 13:23-13:29 UT on 28 August 2023 and is shown in Figure 3a in the main text. The initial distribution of 300 keV electrons at L=1.6 as a function of equatorial pitch angle and longitudes has been added in the supporting information as Figure S7 and shown below:

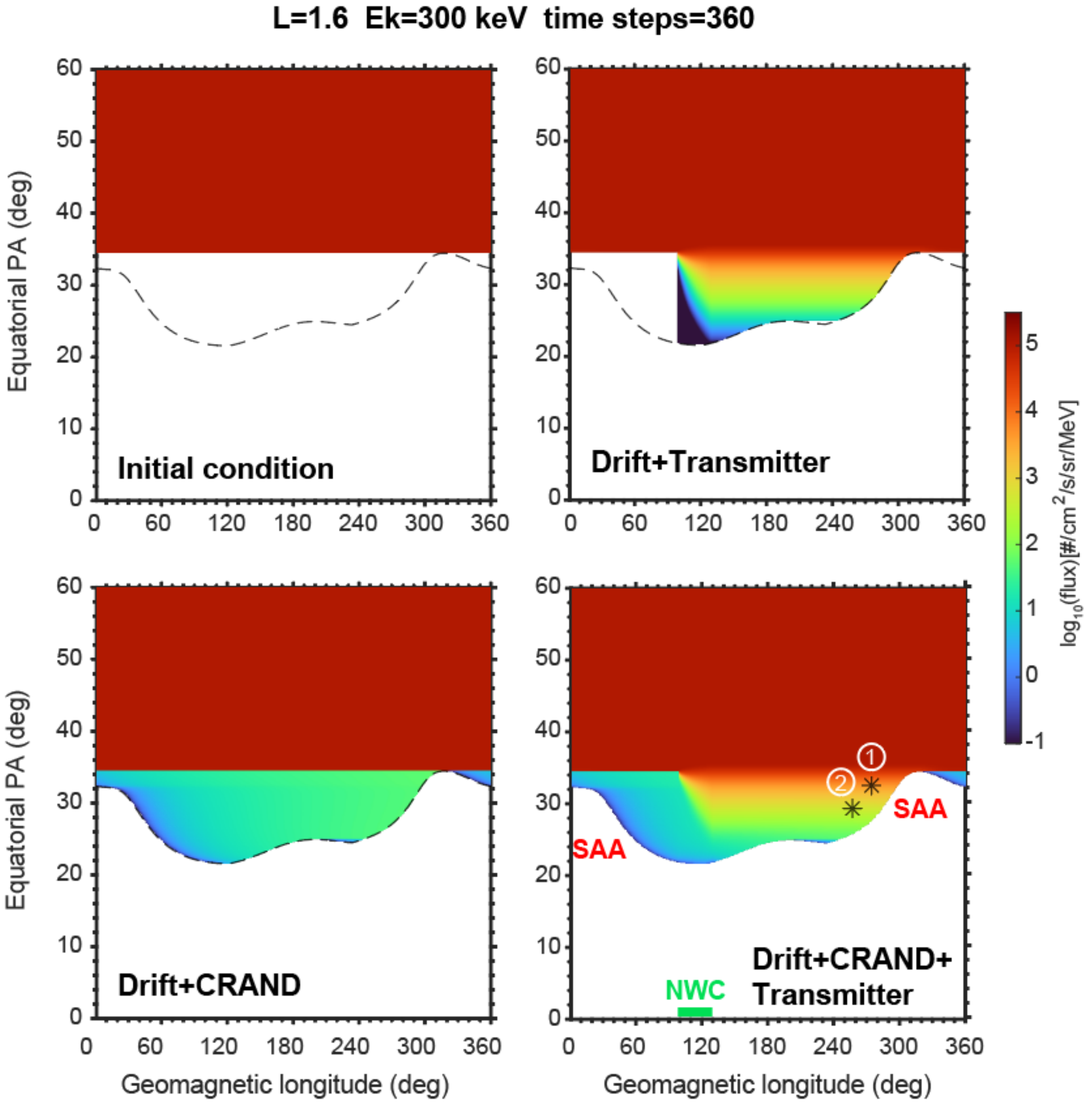


Figure R5. Simulation results of 300 keV electrons at L=1.6 including different physical mechanisms. The simulated electron fluxes are plotted as a function of equatorial pitch angles and longitudes. In this coordinate, electrons drift from left to right and diffuse from higher equatorial pitch angels (PA) to lower ones. The SAA is around [300˚, 20˚] longitude range characterized by larger bounce loss cones indicated by blank space extending to higher equatorial PAs. The longitude range adding pitch angle diffusion coefficients induced by NWC transmitter signals is indicated by the green bar at the bottom of this figure. Electron fluxes corresponding to #1 and #2 asterisks represent simulation results in Figure 3b and 3c, respectively.

*4. How long are run the simulations compared with a drift period? In particular, plotting results in terms of iteration steps is meaningless and should be converted in measure of the drift period.*

**Reply:**

The simulation time is one drift period. In the simulation, one time step equals the time for electrons to drift one degree in geomagnetic longitude. Thus, 360 steps

indicate one drift period. This point has been added in the supporting information.

*5. Which pitch angle range is observed, simulated, and plotted?*

**Reply:**

CIRBE has an active attitude control system to keep the pointing direction of REPTile-2 nearly perpendicular to the background magnetic field with a ~51˚ field of view. Thus, the local pitch angle range of observed electrons is 90˚±25.5˚. In the simulation, electron fluxes in an equatorial pitch angle range [0˚, 90˚] are simulated. When comparing simulation results with observations, the 90˚ local pitch angles convert to equatorial pitch angles based on satellite positions and the IGRF magnetic field model. Two examples of electron equatorial pitch angles measured by CIRBE/REPTile-2 are given as asterisks in Figure S7. The detailed calculation process can be found in Xiang et al. (2019). This point has been added in the text related to Figure S7.

*6. The authors wrote "The Full Diffusion Code (Ni et al., 2008, 2011) is implemented". A code is not "implemented". A method is. Which code is actually used in this study?*

**Reply:**

The Full Diffusion Code is used to calculate pitch angle diffusion coefficients and then the pitch angle diffusion coefficients are used in the Drift-Diffusion-Source model to simulate the wisps. The corresponding sentences have been rephrased in Lines 250-251 as:

"The Full Diffusion Code (Ni et al., 2008, 2011; Shprits & Ni, 2009) is used to compute the bounce-averaged pitch-angle diffusion coefficients based on the quasi-linear theory (Albert, 2007)"

*7. The authors should give the sign convention they use for the harmonics in Eq. (1): is n=1 or n=-1 the dominant one? The current results seem indicate that n=1 is the dominant one, which would be different than the convention usually used in our community. That requires to use an expression for Eq. (1) that is different from the authors' one in the sense that it carries the sign of the gyrofrequency, the latter being evaluated as an absolute value (Albert, 2005).*

**Reply:**

We thank the reviewer for pointing it out. The sentences in Lines 116-118 that "For whistler mode waves, a positive resonance order means the direction of motion of waves and electrons are opposite, while a negative resonance order means the same direction." had indicated that n=1 is the dominant one in equation (1). The equation (1) was used in many previous studies (Koons et al., 1981; Datlowe & Imhof, 1990; Datlowe, 2006; Shen et al. 2022) to discuss the resonance conditions between energetic electrons and VLF transmitter signals. Thus, we keep this equation in the main text. Some explanations regarding equation (1) have been added in Lines 112-113 as:

"Note that Ω doesn't include the sign of the charge so that N=1 is the principal

resonance for whistler mode waves interacting with electrons."

*8. The authors should comment on the results of Cunningham et al. 2021, in particular their Figure 2 and related text.*

**Reply:**

We thank the reviewer for this useful suggestion. Some discussions regarding the Figure 2 of Cunningham et al., (2020) has been added Lines 322-327 as:

"Cunningham et al. (2020) calculate resonance energies of electrons for N=1 and N= -1 at different wave normal angles to explain the observed primary (N=1, parallel waves) and secondary (N= -1, oblique waves) peaks. In this study, we assume the same wave normal angles at different L values and only use different resonance orders (N=-1,1,2) to explain the observed three wisps. The assumed wave normal angles (~65˚) in this study are higher than those (~35˚) in Cunningham et al. (2020)."

*9. Figure 3 of Cunningham et al. 2021 indicates secondary peaks of high flux at higher L-shell than the NWC location which are similar to the one observed here at higher L-shells. This should also be cited.*

**Reply:**

We thank the reviewer for pointing it out. Cunningham et al. (2020) stated that the Figure 3 shows a "diffuse wisp", which is "displaced to lower L, is broader in L, and is more restricted in longitude to near the Western edge of the South Atlantic Anomaly (SAA) that the 'wisps' seen in Sauvaud et al., (2008)". Some flux enhancements can also be observed around 50˚ S latitude in the Figure 3 when comparing panels (b)/(d) with panels (a)/(c). However, these flux enhancements at higher L are more likely due to wave scattering in the slot region rather than generated by NWC transmitter signals. The L values of 500 keV electrons in the third wisps are around L=1.75 (see Figure 2 of the main text) while the flux enhancements in Figure 3 of Cunningham et al. (2020) are close to the outer boundary of the inner belt (~L=2). In addition, multiple wisps are not long-lasting phenomena. Mostly, a single wisp is observed by LEO satellites. Thus, it is hard to observe multiple wisps in the fluxes averaged over a few weeks. The second peaks at higher L are shown in Figure 1 of Cunningham et al. (2020). Thus, we have cited results in Figure 1 of Cunningham et al. (2020) in Lines 318-322 as:

"Using observations from the PROBA-V satellite, Cunningham et al. (2020) reported that the ratio of averaged flux computed with the NWC 'on' versus 'off' has a primary peak at lower L and a secondary peak at higher L. This phenomenon looks like a two-wisp event. Due to the rough energy resolution (seven channels for energy range 0.5-20 MeV), PROBA-V/EPT observations do not show clear wisp structures."

**Finally, we would like to thank the reviewer again for valuable and constructive comments/suggestions, which helped a lot to improve the quality of our paper. We hope that the reviewer will be satisfied with our responses and revisions, and we look forward to hearing from the reviewer soon.**
