# Supplementary material for: Earth‐Based Transmitters Trigger Precipitation of Inner Radiation Belt Electrons: Unveiling Observations and Modeling Results
Source: AGU Adv. 2024 Nov 21;5(6):e2024AV001354. doi: 10.1029/2024AV001354 (PMC11600777; doi:10.1029/2024AV001354)
Supplement: Supplementary file 6 — Author Response to Peer Review Comments [file AGA2-5-0-s007.pdf]

We thank the reviewer for careful reading of the manuscript and the valuable comments for improvements. Accordingly, we have modified the text and added more related references. All the modifications and changes are shown in the revised manuscript as tracked changes. Our responses to the reviewers' comments are listed below. The line numbers in this file correspond to those in the change-tracked file.

Responses to Reviewer #2:

The article "Earth-Based Transmitters Trigger Precipitation of Inner Radiation Belt Electrons: Unveiling Observations and Modeling Results" written by Xiang et al. and submitted to JGR Advances is about observations of electrons flux at LEO which present a typical "wisp" shape. Observations are made from CIRBE, a cube-sat built and managed by the authors' team. This wisp has been observed in the past, first by Sauvaud et al. in 2008 from DEMETER. The novelty is the observation of multiple wisps. These wisps have been attributed to the NWC VLF transmitter signals emitted from Australia in the past. The authors explain the flux by modeling and simulation, with explanations that I had questioned in my last report and which the authors have answer in a very convincing manner.
First, the authors now write clearly they have used the FDC code developed at UCLA by Ni and Shprits in the 2005-2015 years for their computation. This code has been proven to be reliable and trusted since then. This is a relief. Some elements in the Supp. Mat. are minor enough they would let think the authors was learning and developing his model.
Second, the authors provide the 1D plots I had asked of the diffusion coefficients to better understand their claim, which otherwise were not understandable. In these plots newly added in the Supp. Mat. one can understand why they associate the diffusion to a particular harmonic. These figures can also be reproduced for verification purpose. These new results show conclusions can be different according to if the wave propagates in a ducted or unducted mode, as I had pointed out. The authors indeed concluded that too in their rebuttal, a text newly added in the Supp. Mat. The dissociation of the harmonics is also clearly shown while otherwise we were trusting the authors' words without any demonstration.
Third, the authors had not explained enough in their original version the trapping duration (1 drift), the observed pitch angle (low pitch angle at the loss cone) and the initial condition vs pitch angle in a way that one could understand what type of diffusion is discussed and does produce the wisp signature. This is now clarified too.
Fourth, the authors had missed a few important references on this subject. Now, they are cited. As such the various improvements made during revision of this article are significant enough to me that the article becomes convincing and that it can be considered for publication in JGR advances.
Still, I see missing information or important corrections to make. I recommend for now major modification to be done before considering this article for publication. Details are given to the authors below.

Major information and references missing:

1. In the intro, relative to general modelling of NWC power and effects, please refer to Usanova et al. 2022.

Reply:

Usanova et al. (2022) has been cited in Line 106.

2. Please mention the recent observations of wisp signals by ZH-1 in Zhao et al., 2019 (their figure 6, pink box).

Reply:

Zhao et al (2019) has been cited in Line 103.

3. All these studies listed below show diffusion coefficient of VLF transmitter waves with profiles that are peaked in pitch angle due to each of the resonance with VLF transmitter waves. This is known for a long while by any scientists who have done this type of simulations. However, this seems to be discovered here by the authors, reading either their description, with the absence of past references, or naïve explanations in their rebuttals. I ask these references are properly cited with a sentence acknowledging in the main text that fact, something like:

"Multiple past studies have shown that pitch angle diffusion coefficient for VLF transmitter waves have a typical shape composed of a sum of narrow peaks since each principal harmonic creates a peak of significant diffusion for a specific narrow pitch angle range depending on the values of (E, alpha, L) and of n the harmonic number (Abel and Thorne, 1998, 1999; Albert, 2012; Albert et al., 2016, 2020; Ripoll et al. 2014; Ross et al. 2019)".

If the authors prefer to be more specific on where it can be seen in the above references, here is the list of figures: Figure 5 and 8 in Abel and Thorne, 1998; Figure 5 in Abel and Thorne, 1999; Figure 5-6 in Albert, 2012, Figure 1 in Albert et al., 2016; Figure 1-3 in Ripoll et al. (2014); Figure 2 and 5 in Ross et al. 2019; Figure 1 and 2 in Albert et al, 2020.

Reply:

We agree with the reviewer that many past studies have shown that pitch angle diffusion coefficient for VLF transmitter waves have a typical shape composed of a sum of narrow peaks induce by different resonance orders. However, this is not enough to explain multiple wisps observed by CIRBE/REPTile-2. Multiple wisps are quasi-trapped electrons (with pitch angles larger than the local bounce loss cone but smaller than the drift loss cone). These electrons loss into the SAA within one drift period. Therefore, pitch angle diffusion coefficients close to the drift loss cones carry much more weight to determine the flux levels of the wisps. To produce multiple wisps, for electrons with same energies, the pitch angle diffusion coefficients close to the drift loss cone should be dominant by distinct cyclotron resonance orders at different L. As shown in Figure S1 (65° centered wave normal angle):

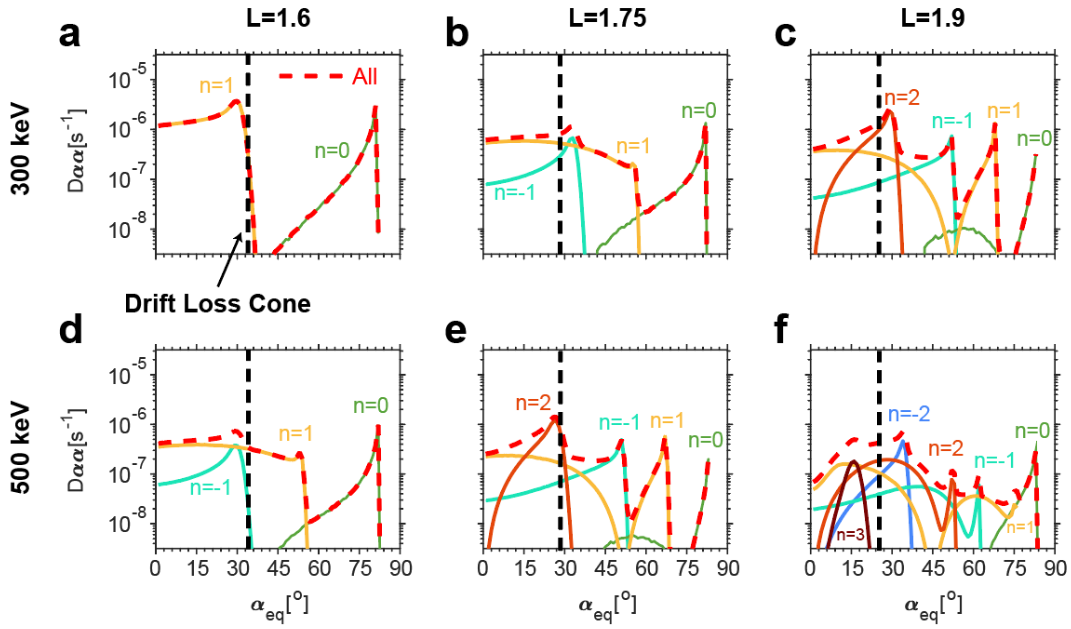

For 300 keV electrons, pitch angle diffusion coefficients close to the drift loss cone (indicated by the vertical dashed line) are dominated by n=1 at L=1.6, n= -1 at L=1.75, and n=2 at L=1.9. Therefore, three wisps are produced. In contrast, as shown in Figure S2 (15° centered wave normal angle):

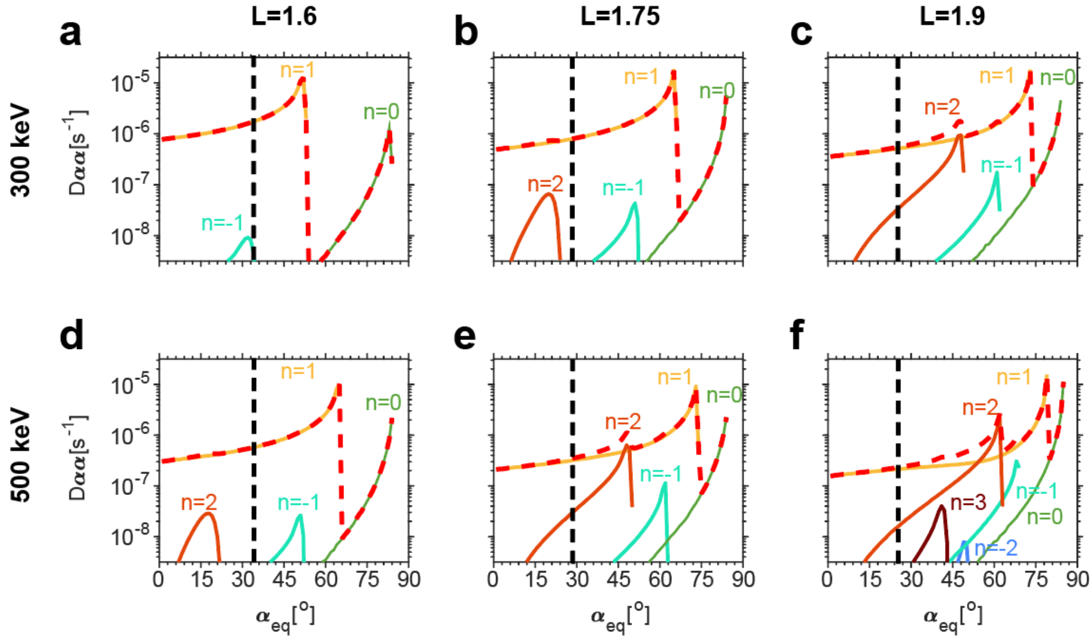

The pitch angle diffusion coefficients of 300 keV and 500 keV electrons at L=1.9 are composed of a sum of several peaks contributed from n=0, n=1, and n=2. However, the pitch angle diffusion coefficients close to the drift loss cone are always dominated by n=1. Thus, only one wisps are produced (see Figure S5).

The list references are helpful to show that different resonance orders produce distinct scattering effects and have been added in Lines 122-126 as:

"Different resonance orders produce distinct scattering effects on electrons, which have been theoretically studied for many years (Lyons et al., 1971, 1974; Abel and

Thorne, 1998, 1999; Ni et al., 2011; Albert, 2012; Albert et al., 2016, 2020; Ripoll et al., 2014; Ross et al., 2019). However, the exact scattering effects from different resonance orders have never been examined by observations.".

4. How do the wisp observations of the authors relate to the observation of zebra stripes in Ukhorskiy et al. 2015 and Lejosne and Roederer 2016. Please cite and discuss the similarities in the text.

Reply:

The similarities/differences between multiple wisps and zebra stripes have been discussed in Lines 356-363 as:

"Similar to multiples wisps, "zebra stripes" in the inner belt also show multiple peaks in the spectrograms of energetic electrons (Ukhorskiy et al., 2015; Lejosne and Roederer, 2016; Li et al., 2024a; Mei et al., 2024). However, there are several differences between the two phenomena: (1) zebra stripes are mainly observed in trapped electrons while multiple wisps are mostly observed in quasi-trapped electrons; (2) the flux peaks of zebra stripes at different L values follow the same drift period of electrons while wisps follow the cyclotron resonance energy curves induced by VLF transmitter signals; (3) the number of flux peaks in zebra stripes increase with time while multiple wisps do not have this temporal feature."

5. In the introduction, about radiation belts, please mention the two recent reviews: Li and Hudson, 2019 and Ripoll et al., 2020

Reply:

The two references have been added in Line 94.

Corrections in the revised text and missing corrections

1. The other reviewer suggested to mention the work of Starks et al 2008 in which smaller amplitudes were computed for wave emitted from the ground. The authors followed his guidance. However, it turns out that Starks et al 2008 made a mistake in their computation using Helliwell attenuation profiles in trans-ionospheric propagation. This was found a few years later by Cohen et al., 2012. This point may have not be known by the other reviewer, and the authors. Therefore, I do not think that a mention to the article of Starks et al. 2008 should be made. If it was still chosen by the authors to refer to Stark et al., 2008, then a mention to the work of Cohen et al. 2012 should be made. I rather recommend not going into that direction and to just erase the following text: "The smaller wave amplitude is estimated based on Starks et al. (2008), which may underestimate the transmitter intensity due to a broad illuminated area, and the peak intensity may be missed by spacecraft. In an updated study…". Then, the authors can simply follow on with their sentence about the Stark et al. 2020 results.

Reply:

We thank the reviewer for pointing it out. The sentences related to Starks et al. (2008) have been deleted. Please see Lines 346-349.

2. Line 113: "Note that Ω doesn't include the sign of the charge so that N=1 is the principal resonance for whistler mode waves interacting with electrons. ". Please indicate more clearly that this is not the convention used in Albert 2005 and Glauert and Horne 2005, which is used by most of the community. Please add the references. Please do not write contraction such as 'doesn't'.

Reply:

The sentences in Lines 113-115 have been rephrased as:

"Note that Ω does not include the sign of the charge so that N=1 is the principal resonance for whistler mode waves interacting with electrons. The convention form of the resonance condition can be found in previous studies (e.g. Albert, 2005; Glauert and Horne, 2005)."

3. Horne et al. 2005 is cited in the reference list but not in the main text. Please fix.

Reply:

Actually, Horne et al. (2005) was cited in Line 97 of the main text.

4. The authors wrote "Considering that the refraction index approaches infinity when the normal angles of highly oblique waves approach the resonance cone angle, we set the pitch angle diffusion coefficient to zero when the refraction index is larger than 200 (Ma et al., 2017).". I agree with the authors on the method but I do not see that point being mentioned in Ma et al., 2017. Could the authors check? Also Mourenas, Artemeiv and co-authors have published a series of articles between 2013-2017 in which large refraction indices were considered to lead to large diffusion coefficients. In other words, highly oblique waves would lead to strong pitch angle diffusion. This was based on wrong approximative maths supported by a wrong code, as shown and discussed in a dedicated article (Albert, 2017). The correct result is: when refraction index approaches infinity (for highly oblique waves) the pitch angle diffusion coefficient tends to zero, as the authors do consider in their article. I recommend the authors cite the author who found the right result first. This is Albert, 2017. In simple words, I recommend to replace the citation Ma et al., 2017 by Albert, 2017.

Reply:

We thank the reviewer for pointing it out. The reference Ma et al. (2017) has been replaced by Albert, (2017). Please see Line 271.

5. Line 324: In "In this study, we assume the same wave normal angles at different L values and only use different resonance orders (N=-1,1,2) to explain the observed three wisps.", it is unclear if 'this' refers to the authors' study or Cunningham et al. Please use 'our' instead.

Reply:

Thanks, "In this study" has been changed to "In our study". Please see Line 328.
